# Aerosols and hydrocarbons in the atmosphere of a white dwarf planet

**DOI:** 10.1038/s41586-026-10514-7

**Published:** 2026-07-01

**Authors:** Ryan J. MacDonald, Christopher E. O’Connor, Victoria A. Boehm, E. M. May, David K. Sing, Elijah Mullens, L. C. Mayorga, Trevor O. Foote, Simon Blouin, Logan A. Pearce, Nikole K. Lewis, Jeff Valenti, Natasha E. Batalha, Maura Lally, Joshua D. Lothringer, Mark S. Marley, Ishan Mishra, Susan E. Mullally

**Affiliations:** 1 University of St Andrews, St Andrews, UK; 2 University of Michigan, Ann Arbor, MI USA; 3 Cornell University, Ithaca, NY USA; 4 Northwestern University, Evanston, IL USA; 5 Johns Hopkins Applied Physics Laboratory, Laurel, MD USA; 6 Johns Hopkins University, Baltimore, MD USA; 7 NASA Goddard Space Flight Center, Greenbelt, MD USA; 8 University of Victoria, Victoria, British Columbia Canada; 9 Steward Observatory, University of Arizona, Tucson, AZ USA; 10 Space Telescope Science Institute, Baltimore, MD USA; 11 NASA Ames Research Center, Moffett Field, CA USA; 12 Lunar and Planetary Laboratory, University of Arizona, Tucson, AZ USA; 13 Jet Propulsion Laboratory, California Institute of Technology, Pasadena, CA USA

**Keywords:** Exoplanets, Stellar evolution, Atmospheric chemistry, Giant planets

## Abstract

Most stars, including our Sun, will one day evolve into red giants and, subsequently, white dwarfs. Several planet candidates have recently been identified orbiting white dwarfs^1–4^, demonstrating that planets can survive the stellar post-main-sequence stage intact. Little is known about the atmospheric composition of post-main-sequence planets, with the most evolved transiting planets with atmospheric detections so far orbiting subgiants^5,6^. Here we report an atmospheric detection for the white dwarf planet WD 1856 b, achieved through transmission spectroscopy with the James Webb Space Telescope (JWST) Near-Infrared Spectrograph (NIRSpec) PRISM. Our 0.5–5.0-μm spectrum reveals the presence of hydrocarbons (odds ratio of 167:1–5,377:1, with CH_4_ preferred at 17:1–30:1), aerosols (2 × 10^5^:1–2 × 10^6^:1) and thermal emission from the planetary nightside (2 × 10^63^:1–2 × 10^73^:1). Our spectral analysis constrains the mass of WD 1856 b to 4.3–10.9 *M*_J_, finds a carbon-enriched atmosphere (with a CH_4_ abundance of approximately 7%) and an effective temperature exceeding the expected planetary equilibrium temperature (390–412 K versus 160 K). On the basis of cooling models, these results indicate that WD 1856 b underwent a migration-related reheating event 3.0–5.5 Gyr into the white dwarf phase, consistent with post-main-sequence tidal evolution to the present-day 0.02-au circular orbit. Our results provide a window into the ultimate fate of giant planets orbiting stars with masses similar to our Sun.

## Abstract (web summary)

Transmission spectroscopy of the planet WD 1856 b, orbiting a white dwarf, with the James Webb Space Telescope reveals the presence of hydrocarbons and aerosols in its atmosphere, providing insight into its mass and heating history.

## Main

We observed a transit of WD 1856 b on 27 April 2023 with JWST’s NIRSpec instrument, using the PRISM mode, as part of Guest Observer Program 2358 (PI: Ryan MacDonald). WD 1856 b is a cool (*T*_eq_ = 160 K), Jupiter-sized (0.9 *R*_J_) planet transiting the white dwarf WD 1856+534 (ref. ^1^). The host white dwarf (*T*_eff_ = 4,710 K, 0.0131 *R*_Sun_, 0.518 *M*_Sun_, *t*_cool_ ≈ 6 Gyr (ref. ^1^)) is 25 pc from Earth and has a DA spectral class^7^. The current close-in orbit of WD 1856 b (0.02 au/1.4 days) requires orbital evolution after the main sequence to avoid engulfment during the red giant phase. Hypotheses for the orbital evolution of WD 1856 b include common-envelope evolution during the red giant or asymptotic giant branch (AGB) phase^8,9^ or, alternatively, high-eccentricity migration^10^. Distinguishing these scenarios has proved challenging, given the uncertain planetary mass^1,11,12^. As the host white dwarf WD 1856 is a relatively faint target (*J* = 15.677), we gave priority to the wide wavelength range (0.6–5.5 μm at a mean resolving power of *λ*/Δ*λ* ≈ 100) and high throughput of the NIRSpec PRISM over other JWST instrument modes. Our observation lasted 1.98 h, of which the transit of WD 1856 b lasted 8 min.

**Fig. 1:**
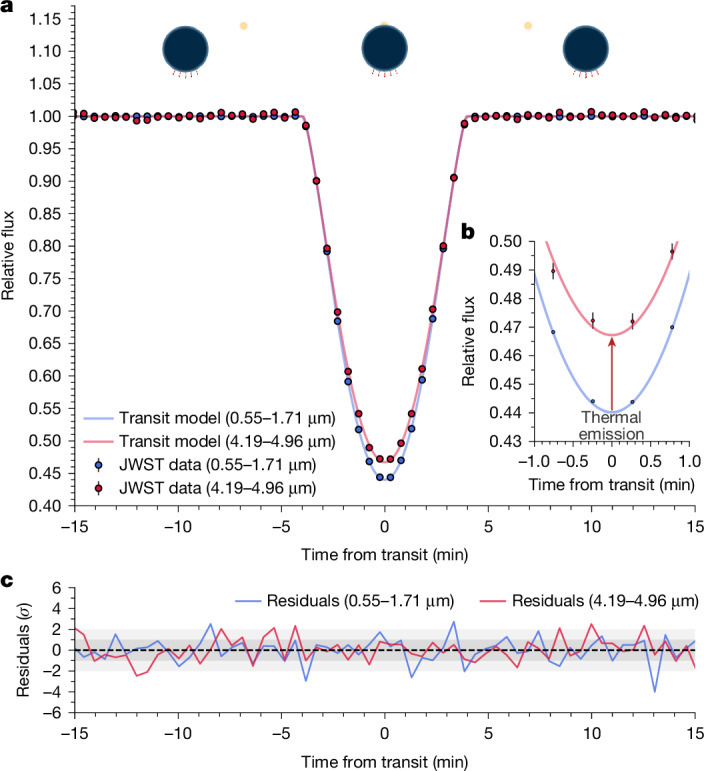
Detection of nightside thermal emission from the atmosphere of WD 1856 b. **a**, JWST NIRSpec PRISM transit observations of the white dwarf planet WD 1856 b integrated over two broadband wavelength regions: 0.55–1.71 μm (blue data points) and 4.19–4.96 μm (red data points). The best-fitting model transit light curves are overplotted (blue and red curves). The transit event is shallower at longer wavelengths owing to planetary thermal emission diluting the transit depth. The top schematic depicts the grazing transit geometry of the WD 1856 system, with the planet and white dwarf shown to scale. **b**, Zoom-in of the mid-transit to demonstrate the high signal-to-noise detection of the nightside thermal emission. The 1*σ* errors for the blue data points are too small to be seen (the mean 1*σ* errors are 5.3 × 10^−4^ from 0.55–1.71 μm and 2.8 × 10^−3^ from 4.19–4.96 μm). **c**, Residuals between the data and best-fitting model. Source data

**Fig. 2:**
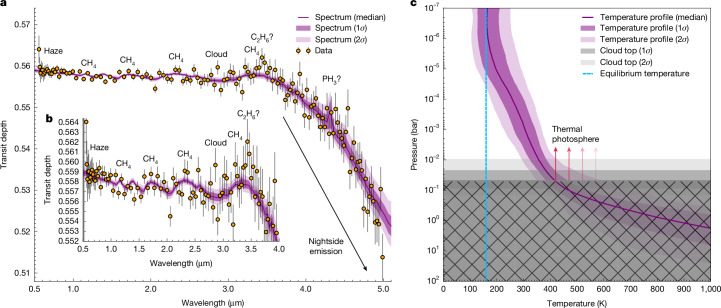
Atmospheric retrieval of the transmission spectrum of WD 1856 b. **a**, The JWST NIRSpec PRISM transmission spectrum of WD 1856 b (FIREFLy data reduction; orange circles with 1*σ* error bars) is compared with the retrieved model spectrum (purple line and contours, showing the median, 1*σ* and 2*σ* credible intervals). Several absorption features from CH_4_ are detected, alongside nightside thermal emission, continuum aerosol opacity and tentative evidence of PH_3_ and C_2_H_6_. **b**, Zoom-in to highlight the short-wavelength spectral features. **c**, Retrieved temperature profile (purple contours) and cloud-top pressure (grey contours) from the transmission spectrum of WD 1856 b. An optically thick cloud deck near 100 mbar (grey hatching) blocks thermal emission from deeper layers. The top of the hatched region corresponds to the median retrieved cloud-top pressure and the 1*σ* and 2*σ* credible regions appear above (horizontal grey shading). Most thermal emission arises from the cloud-top pressure near 100 mbar (red arrows). Source data

We reduced the JWST observations using two independent data pipelines, FIREFLy^13^ and Juniper (newly presented here; Methods). Each reduction pipeline yielded sets of spectroscopic transit light curves for WD 1856 b, which we independently fit with a transit model (Methods). Because most existing stellar model grids do not include models for evolved or remnant objects, our transit models used a custom limb-darkening prescription for WD 1856 derived from a white dwarf model^14^ fitted to the out-of-transit host flux (Methods). Figure 1 shows transit light curves of WD 1856 b from FIREFLy, integrated over two broadband wavelength regions from 0.55–1.71 μm and 4.19–4.96 μm. Our JWST observations find a 3% shallower transit depth in the second, near-infrared, wavelength region compared with visible wavelengths. The physical mechanism for this transit depth difference is planetary thermal emission, as we establish below. Our PRISM transmission spectrum of WD 1856 b from the FIREFLy code is shown in Fig. 2a,b.

We model the transmission spectrum of WD 1856 b using the radiative transfer and retrieval code POSEIDON^15,16^, adapted here to include partial planet–star overlap (that is, grazing transit geometry) and the effect of nightside thermal emission^17,18^ (Methods). We consider the main carbon, oxygen, nitrogen, sulfur and phosphorous carriers expected in a H_2-_dominated atmosphere at the equilibrium temperature of WD 1856 b and several disequilibrium tracers, resulting in the following gases: CH_4_, NH_3_, H_2_O, CO_2_, CO, C_2_H_2_, H_2_S, PH_3_, HCN, C_2_H_4_ and C_2_H_6_. Our retrievals freely fit the temperature structure, chemical composition, an opaque cloud deck and a power law haze^15^. Given the uncertain mass of WD 1856 b, our retrievals fit the mass jointly with the atmospheric properties. We conducted retrievals on both data reductions, finding excellent consistency between FIREFLy and Juniper (Methods). The retrieved spectrum and temperature structure for the FIREFLy reduction are shown in Fig. 2.

**Fig. 3:**
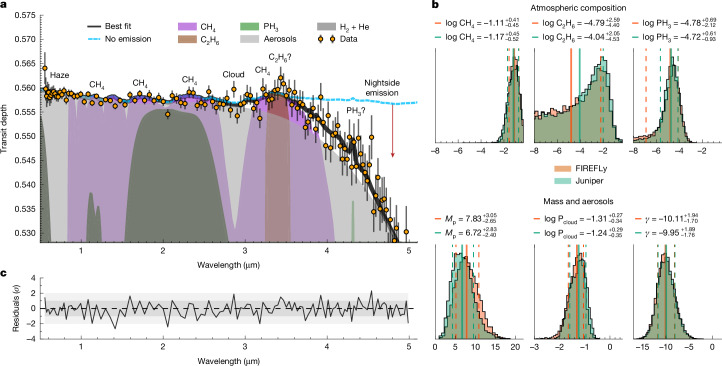
Best-fitting spectrum and atmospheric properties of WD 1856 b. **a**, Spectral decomposition illustrating the model components required to explain the JWST transmission spectrum of WD 1856 b (FIREFLy reduction; orange circles with 1*σ* error bars). The best-fitting model (black curve) contains absorption from several CH_4_ features (purple shading), H_2_ + He continuum absorption (dark grey shading) and tentative evidence of C_2_H_6_ near 3.4 μm (brown shading) and PH_3_ near 4.3 μm (green shading). Aerosol continuum opacity (light grey shading) blocks thermal emission from the deep atmosphere in windows between CH_4_ bands, obscuring the ‘negative spikes’ that would otherwise be observed near 0.7 μm, 2.9  μm and longwards of 4.0 μm for a clear atmosphere. The best-fitting model without nightside thermal emission (dashed blue curve) cannot explain the much lower transit depths longwards of 3.5 μm. **b**, Retrieved atmospheric properties, planetary mass and aerosol properties from the transmission spectrum of WD 1856 b for the FIREFLy (orange histograms) and Juniper (green histograms) data reductions. The median (solid lines) and 1*σ* credible intervals for each parameter (dashed lines) are overlaid. **c**, Residuals between the data and best-fitting model.

Our retrievals establish that at least one hydrocarbon ($4.5\sigma /\mathrm{ln}{\mathcal{B}}\,=$ $8.59$ for Juniper, $3.6\sigma /\mathrm{ln}{\mathcal{B}}=5.12$ for FIREFLy) is present in the atmosphere of WD 1856 b. The main contributor to the hydrocarbon inference is CH_4_ ($3.1\sigma /\mathrm{ln}{\mathcal{B}}=3.40$ for Juniper, $2.9\sigma /\mathrm{ln}{\mathcal{B}}=2.87$ for FIREFLy) but C_2_H_6_ also contributes to the best-fitting model. Figure 3a,c shows spectral contributions to our best-fitting model, which achieves an excellent fit to the FIREFLy data (${\chi }_{\nu }^{2}=1.03$ with 102 degrees of freedom). The fit quality is slightly less good for Juniper (${\chi }_{\nu }^{2}=1.28$, indicating consistency with the data within 2*σ* for 102 degrees of freedom) but the retrieval results are nonetheless consistent between the two data reductions. CH_4_ causes three prominent absorption bands near 1.75 μm, 2.3 μm and 3.3 μm, with the retrieved CH_4_ abundance (2–20%) indicating an atmospheric metal enrichment of about 100 × solar ($\log ({\rm{C}}/{\rm{H}})=2.2{4}_{-0.40}^{+0.33}$ for FIREFLy and $2.2{1}_{-0.44}^{+0.35}$ for Juniper in solar units, with corresponding 3*σ* lower limits of 10.8 × solar and 7.9 × solar, respectively). We confirmed that the CH_4_ inference arises from several bands by repeating a FIREFLy retrieval with the data from 3.1–3.5 μm masked, yielding a 3.0*σ* model preference. The best-fitting model includes a contribution from PH_3_ near 4.3 μm, which could be an indicator of disequilibrium vertical mixing as in the upper atmosphere of Jupiter^19^. However, model degeneracies and lower signal-to-noise at longer wavelengths preclude a detection of PH_3_ with the present data. Similarly, a feature attributable to C_2_H_6_ at 3.4 μm is partially degenerate with CH_4_ absorption near 3.3 μm, so C_2_H_6_ is not significantly detected. However, the combined evidence for any hydrocarbon (that is, CH_4_, C_2_H_2_, C_2_H_4_ and C_2_H_6_) is statistically significant at approximately 4*σ*. No other chemical species (for example, NH_3_ or H_2_O) are detected (see Methods and Extended Data Fig. 4 for upper limits).

Besides hydrocarbons, we detect thermal emission from the observer-facing nightside hemisphere ($18.5\sigma /\mathrm{ln}{\mathcal{B}}=169$ for Juniper and $17.3\sigma /\mathrm{ln}{\mathcal{B}}=146$ for FIREFLy) and aerosols ($5.7\sigma /\mathrm{ln}{\mathcal{B}}=14.5$ for Juniper and $5.3\sigma /\mathrm{ln}{\mathcal{B}}=12.3$ for FIREFLy). Emission from the nightside hemisphere (about 400 K) causes the substantial downwards slope longwards of 3.5 μm. Because thermal emission in opacity windows arises from deeper, hotter layers, an optically thick cloud deck must exist for pressures deeper than roughly 10 mbar (Fig. 2c) to explain the lack of ‘negative spikes’ between the CH_4_ absorption bands (Fig. 3a,c). Because most thermal emission arises from the cloud-top temperature, the recent measurement of an approximately 190 K thermal excess in MIRI photometry of the WD 1856 system^12^ (at a different epoch near quarter phase) potentially indicates variable cloud-top pressures for different hemispheres or in time. Alternatively, an unidentified systematic or calibration issue affecting either instrument may reconcile the difference, but we did not identify any such effect in either dataset. Further evidence of aerosols arises from the scattering slope shortwards of 1 μm, which is more prominent than H_2_ Rayleigh scattering from a clear atmosphere. We investigated several specific aerosols using a Mie scattering model, but the present data are insufficient to identify the chemical composition of the aerosol (Methods). Finally, we report the first constrained measurement of the mass of WD 1856 b: 4.3–10.9 *M*_J_ (1*σ* spread across both data reductions or $6.{7}_{-2.4}^{+2.8}\,{M}_{{\rm{J}}}$ for FIREFLy and $7.{8}_{-2.7}^{+3.1}\,{M}_{{\rm{J}}}$ for Juniper). Our mass constraint arises from a scale height trade between the CH_4_ absorption features and the integrated optical depth to the emitting pressure of the nightside atmosphere.

The atmospheric temperature at which WD 1856 b radiates to space (*T*_eff_ = 390–412 K, or $40{0}_{-10}^{+6}\,{\rm{K}}$ for FIREFLy and $40{5}_{-11}^{+7}\,{\rm{K}}$ for Juniper; Methods) is substantially increased over both the equilibrium temperature (160 K) and that expected for an evolved giant planet at the roughly 10 Gyr system age (≲100 K)^1^. Given the retrieved mass and the present-day circular orbit, internal power sources such as deuterium fusion or tidal heating cannot contribute to the observed effective temperature (Methods). However, a thermal reset of WD 1856 b can have occurred by either tidal heating during high-eccentricity migration^10^ or immersion in the stellar envelope during a common-envelope phase^8,9^. These two scenarios predict different migration times relative to the death of the host star: common-envelope evolution coincides with the end of the AGB phase of the progenitor (5.4 ± 0.7 Gyr ago; Methods) and lasts about 1 Myr (ref. ^20^), whereas high-eccentricity migration can occur any time during the white dwarf phase. Because substellar objects cool down at a predictable rate, our planetary mass and temperature constraints provide critical information to infer the reheating epoch.

**Fig. 4:**
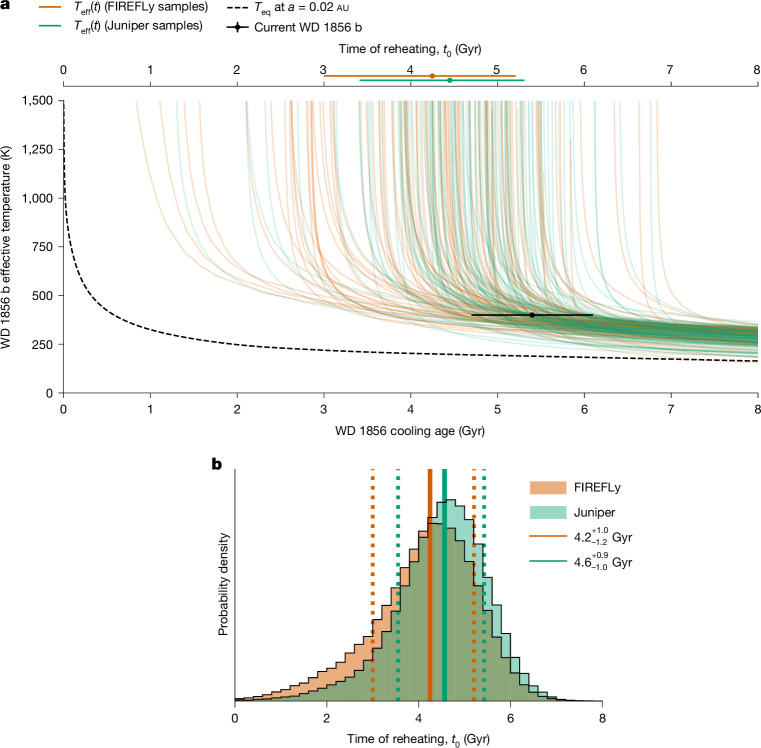
Evolutionary history of WD 1856 b. **a**, Reconstructed thermal evolution of WD 1856 b. An ensemble of randomly drawn thermal histories compatible with the JWST-derived constraints on the mass and effective temperature of WD 1856 b and the cooling age of the host white dwarf are shown (orange curves for the FIREFLy data, green curves for Juniper). The thermal history for an object at the 0.02-au orbit of WD 1856 b maintaining the zero-albedo equilibrium temperature is overlaid for comparison (dashed black curve). **b**, Inferred host cooling age at the time of reheating of WD 1856 b during migration, *t*_0_, based on the ensemble of reconstructed thermal histories (orange and green histograms for FIREFLy and Juniper, respectively). The median (solid line) and 1*σ* credible interval (dashed lines) are overlaid and also plotted above panel **a**. Because common-envelope evolution predicts *t*_0_ consistent with zero, the present-day mass and temperature favour migration billions of years after the conclusion of the AGB phase.

We reconstructed the thermal evolution of WD 1856 b using theoretical cooling models for substellar objects (Methods) to extrapolate its effective temperature backwards from the present. Figure 4 shows a random set of reconstructed thermal histories for WD 1856 b, alongside the equilibrium temperature evolution (tracing the cooling of the white dwarf). The range of possible histories is governed by uncertainties on the mass of WD 1856 b and the cooling age of the host. For each thermal history, we calculated the cooling age of the white dwarf at the time of reheating (denoted *t*_0_; Methods). In Fig. 4, *t*_0_ corresponds roughly to the time when each cooling model intercepts the top of the diagram. We find that the reheating of WD 1856 b occurred 3.0–5.5 Gyr after the end of the AGB phase ($4.{2}_{-1.2}^{+1.0}\,{\rm{Gyr}}$ for FIREFLy and $4.{6}_{-1.0}^{+0.9}\,{\rm{Gyr}}$ for Juniper; Fig. 4b). More conservative 2*σ* lower bounds on *t*_0_ imply that reheating occurred at least 1.4 Gyr (FIREFLy) or 2.1 Gyr (Juniper) after the AGB phase. The AGB and post-AGB/pre-white-dwarf phases are extremely brief by comparison, lasting less than 2 Myr and 0.1 Myr, respectively (Methods). The timing of the inferred reheating event is, therefore, inconsistent with common-envelope evolution during either phase. Therefore, WD 1856 b most likely underwent high-eccentricity migration to its current orbit, with the inferred reheating event corresponding to tidal circularization.

WD 1856 b represents the first well-characterized transiting planet orbiting a white dwarf. The inferred thermal evolution of WD 1856 b demonstrates that high-eccentricity migration is a plausible fate for giant planets after the stellar main sequence. The retrieved CH_4_ abundance is similar to the deep atmosphere of Neptune (4% (ref. ^21^)), which requires notable carbon enrichment of the planet’s H_2_ envelope from volatile-rich material, whether accreted before its migration^22–24^ or afterwards^25^. This high atmospheric metallicity (≳100 × solar) enhances aerosol production^26–28^, consistent with the detection of a short-wavelength scattering slope in our transmission spectrum. As WD 1856 b demonstrates, spectroscopy of planets orbiting white dwarfs offers a new opportunity to determine the fate of planetary systems after the death of their star.

## Methods

### Data reduction

Our PRISM observation used 33 groups across 240 integrations, with each integration lasting 29.8 s, for a total observing time of 1.98 h. Although the transit duration of WD 1856 b only lasts 8 min, we selected this observing window to ensure JWST captured a transit of WD 1856 b with sufficient out-of-transit baseline for detector settling. We used two independent codes to reduce the NIRSpec PRISM observation of WD 1856 b and extract transmission spectra: FIREFLy and Juniper. Here we detail the reduction and light curve fitting approach used by each code.

#### FIREFLy

We first reduced the WD 1856 b data using the Fast InfraRed Exoplanet Fitting Lyghtcurve (FIREFLy)^13,29,30^ reduction suite. The reduction started with the uncalibrated (uncal.fits) images and a customized jwst pipeline reduction. During stages 1 and 2, the 1/*f* noise was removed at the group level, using the top and bottom six rows to measure the count level for each column and subtracting the median value. The dark current step was skipped and the jump step was performed with a rejection threshold of 20. During stage 3, we then used the custom-run pipeline 2D images after the jwst.assign_wcs step and performed customized cleaning of bad pixels, cosmic rays and hot pixels. We used cross-correlation to measure the positional shift of the spectral trace across the detector and shift-stabilized the images with flux-conserving interpolation. This procedure has been found to reduce the amplitude of position-dependent systematic trends^13,29^. An aperture size of 5.7 pixels was used to extract the spectra, with this product used to fit the transit light curves and extract the exoplanet spectra.

**Extended Data Fig. 1:**
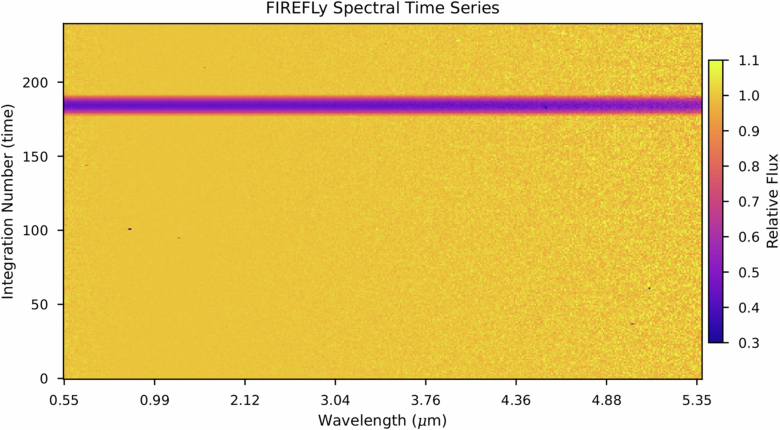
NIRSpec PRISM light curves for the transit of WD 1856 b. The spectrophotometric transit light curves from the FIREFLy data reduction show the relative flux of the WD 1856 system as a function of wavelength and time.

During stages 4 and 5, we fit the white light curve using a linear baseline and a limb-darkened transit model^31^. The stellar limb darkening was modelled with the procedures from ref. ^32^ and a quadratic function using ExoTiC-LD^33^. The values were fixed to the best-fitting theoretical host white dwarf model values (see the ‘White dwarf host spectrum’ section below). We measured the white light curve using the 0.55 to 3 μm region, such that the planetary emission would not bias the transit depth and resulting system parameters. The NIRSpec PRISM spectral time series for FIREFLy is shown in Extended Data Fig. 1). Several pre-transit exposures showed abnormally low flux levels, which we flagged as outliers and removed from the remaining analysis. These outliers seem to be because of clusters of bright/hot pixels, so are probably associated with snowball events. We fixed the period using the results from ref. ^34^. The best-fit white light curve system values are given in Extended Data Table 1. The transmission spectral light curves used 3-pixel binning and were fit with the same model, setting the system parameters to be fixed to the white light curve values.

#### Juniper

We also applied Juniper, a new custom pipeline for JWST NIRSpec observations, to reduce our WD 1856 b data. Juniper contains a wrapper for stages 1 and 2 of the jwst pipeline with custom steps. We start our processing from Juniper stage 1 with the uncal.fits files from the Mikulski Archive for Space Telescopes (MAST). We opt to disable the jwst stage 1 jump detection step and instead handle cosmic rays through custom procedures in later stages. Before ramp fitting, group-level background subtraction is performed using the top and bottom six rows as the background region to reduce scatter in the extracted light curves. We spatially filter 3*σ* outliers from this region and average along columns to determine the background level of counts per column, which is subtracted from the full group. We then proceed with jwst stage 1 ramp fitting and gain scaling. Juniper stage 2 is a pure wrapper for jwst stage 2; we carry out this stage with the flat field and photom steps disabled, as neither is required to measure transit depth and we observed the former to increase the noise in the spectral extraction.

Juniper stage 3 performs extra cleaning at the integration level. We first mask pixels flagged by the jwst pipeline for data quality issues. We then reject cosmic rays in time over two iterations, replacing 6.5*σ* outliers with the median value of the pixel in time. Finally, a second round of background subtraction, using the same strategy as the group-level background subtraction in stage 1, is performed using the top and bottom three rows with outliers masked at 3*σ*.

Stage 4 of the Juniper pipeline extracts 1D spectra, which are subsequently binned to produce light curves. Our aperture is centred on the brightest row of the trace and extends ±3 pixels above and below it. We perform optimal extraction^35^ to extract the 1D spectrum, taking as our extraction profile the median image of the trace contained in the aperture, normalized along columns. We sum across all pixels from 0.552 to 3 μm to extract a broadband light curve; we choose not to include light from wavelengths longer than 3 μm as this light is strongly affected by contamination from nightside thermal emission, which affects the determined system parameters (for example, semimajor axis, transit epoch, impact parameter). We then bin every 3 pixels to produce 137 median-normalized spectroscopic light curves at nearly native resolution, spanning 0.552 to 5.360 μm. Our extracted broadband and spectroscopic light curves would typically be sigma-clipped to further remove outliers; however, this technique is prone to clipping out the transit itself owing to the short transit duration and large transit depth. We therefore disable this procedure and use alternative outlier rejection procedures in stage 5.

Juniper stage 5 is the final stage of the pipeline, which fits transit models to each light curve to extract transit depth and produce a transmission spectrum. Our fitting procedure is a two-step process combining linear and nonlinear fitting techniques, which we use to clean outliers that sigma-clipping cannot safely remove. We start by using a linear least-squares estimator to fit a batman transit model^31^, applying a quadratic limb-darkening law with coefficients generated with ExoTiC-LD^33^ using a custom white dwarf model produced by fitting a white dwarf spectrum to the out-of-transit flux of WD 1856 (described below). Our model is further multiplied by a linear-in-time trend to account for visit-long ramp effects. We then compute the residuals of the fitted transit and systematics model and clip any points in the light curve that produce 3*σ* outliers in the residuals. We compute the standard deviation of the sigma-clipped residuals to estimate the photometric uncertainty, which we supply to the next step of our fit procedure. We refit the sigma-clipped light curve using Markov chain Monte Carlo methods^36^ to extract our final planet–star radius ratio spectrum. We first fit our 0.552 to 3 μm broadband light curve using this two-step fitting process to determine the broadband depth, semimajor axis *a*/R_*_, inclination *i*, mid-transit epoch *t*_C_ and linear-in-time systematics model parameters, from which we derive the impact parameter *b* and its uncertainty. The orbit period *P* was held fixed to 1.407939217 days based on a follow-up paper studying transit timing variations in the WD 1856+534 system^34^. Our broadband light curve analysis yielded *a*/R_*_ = 339.25 ± 5.92 and *b* = 7.34 ± 0.20. We then fix these values as well as *t*_C_ as determined by the broadband light curve fit for all subsequent spectroscopic light curve fits. We fit every spectroscopic light curve with our two-step process to determine *R*_p_(*λ*)/R_*_ and the linear systematics trend in every wavelength channel. Our broadband light curve fit achieves residuals of 522 ppm, whereas our spectroscopic fits achieve median residuals of 8,153 ppm. We present our fitted system parameters (*a*/R_*_, *b*, *R*_p_/R_*_) and broadband transit depth in Extended Data Table 1.

### Grazing transit spectroscopy

The unique transit geometry of the WD 1856 system required us to develop a new approach to express and model transmission spectra. A transmission spectrum encodes the wavelength-dependent effective area of a planet relative to its host star. Exoplanet analyses typically take the spectroscopic planet–star radius ratio from light curve fits, *R*_p_(*λ*)/R_*_, and then express the transmission spectrum as ${R}_{{\rm{p}}}{(\lambda )}^{2}/{R}_{\ast }^{2}$. This quantity is equivalent to the transit depth for a planet with radius *R*_p_(*λ*) fully occulting a non-limb-darkened star of radius R_*_. However, because WD 1856 b is seven times larger than its white dwarf host with a grazing transit, the transmission spectrum cannot be written as ${R}_{{\rm{p}}}{(\lambda )}^{2}/{R}_{\ast }^{2}$. Indeed, the transit depth of WD 1856 b is dependent on time throughout the transit (see ref. ^11^), with a maximum transit depth corresponding to the time of greatest areal overlap between the planet and its host (Fig. 1). We therefore express the transmission spectrum of WD 1856 b as the wavelength-dependent maximum transit depth at the time of mid-transit, *A*_p_/A_*_.

We convert the spectroscopic planet–host radius ratio into the mid-transit transit depth by calculating the time-dependent area overlap of two discs. The overlapping area of two circles with radii *R*_p_ (representing the planet) and R_*_ (representing the white dwarf), separated by a distance *d*, is given by:

1 $${A}_{{\rm{p}}}(d)={R}_{{\rm{p}}}^{2}\theta +{R}_{\ast }^{2}\phi -\frac{1}{2}{R}_{{\rm{p}}}^{2}\sin (2\theta )-\frac{1}{2}{R}_{\ast }^{2}\sin (2\phi )$$

in which

2 $$\theta ={\cos }^{-1}\,\left(\frac{{d}^{2}+{R}_{{\rm{p}}}^{2}-{R}_{\ast }^{2}}{2d{R}_{{\rm{p}}}}\right)$$

3 $$\phi ={\cos }^{-1}\,\left(\frac{{d}^{2}+{R}_{\ast }^{2}-{R}_{{\rm{p}}}^{2}}{2d{R}_{\ast }}\right)$$

Considering the time of mid-transit (when *d* = bR_*_, in which *b* is the transit impact parameter), we can express the maximum transit depth as:

4 $$\frac{{A}_{{\rm{p}}}}{{A}_{\ast }}=\frac{1}{{\rm{\pi }}}\left[{\left(\frac{{R}_{{\rm{p}}}}{{R}_{\ast }}\right)}^{2}\left(\theta -\frac{1}{2}\sin 2\theta \right)+\left(\phi -\frac{1}{2}\sin 2\phi \right)\right]$$

in which

5 $$\theta ={\cos }^{-1}\,\left[\frac{{b}^{2}+{\left(\frac{{R}_{{\rm{p}}}}{{R}_{\ast }}\right)}^{2}-1}{2b\left(\frac{{R}_{{\rm{p}}}}{{R}_{\ast }}\right)}\right]$$

6 $$\phi ={\cos }^{-1}\,\left[\frac{{b}^{2}-{\left(\frac{{R}_{{\rm{p}}}}{{R}_{\ast }}\right)}^{2}+1}{2b}\right]$$

We use equations (4), (5) and (6) to map the spectroscopic radius ratio, *R*_p_/R_*_, and impact parameter from the spectroscopic light curve fits of each data reduction into the equivalent mid-transit transmission spectrum. We use the uncertainties Python package to propagate errors using these formulae. This approach automatically removes offsets between the different reductions for *R*_p_/R_*_, as each corresponding pair of *R*_p_/R_*_ and *b* must yield consistent *A*_p_/A_*_ to have the same transit shape (that is, to match Fig. 1).

**Extended Data Fig. 2:**
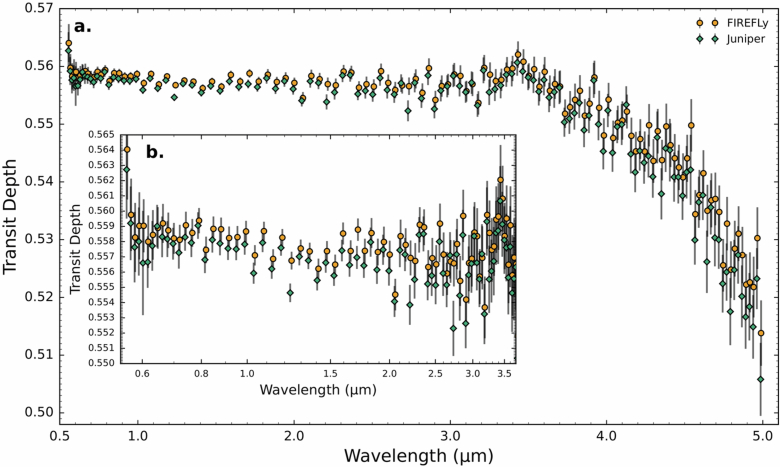
Comparison of transmission spectrum data reductions. **a**, The WD 1856 b transmission spectrum, expressed as the ratio between the mid-transit planet area occulting the host and the host surface area (*A*_p_/A_*_), is shown for FIREFLy (orange circles with 1*σ* error bars) and Juniper (green diamonds with 1*σ* error bars). **b**, Zoom-in on shorter wavelengths. Source data

We show our final transmission spectra of WD 1856 b, expressed as the mid-transit transmission spectrum (*A*_p_/A_*_), in Extended Data Fig. 2. Both reductions clearly detect the strong signature of nightside contamination (the slope to lower transit depths at longer wavelengths) and lead to consistent atmospheric inferences from our retrieval analysis (see the ‘Atmospheric retrieval analysis’ section). We note that the two reductions partially deviate at wavelengths longer than 5 μm—mainly because of differences in the light-darkening treatments and uncertainties in the red edge detector behaviour—so we restricted our atmospheric analysis for WD 1856 b to the NIRSpec PRISM data from 0.5–5 μm.

### White dwarf host spectrum

**Extended Data Fig. 3:**
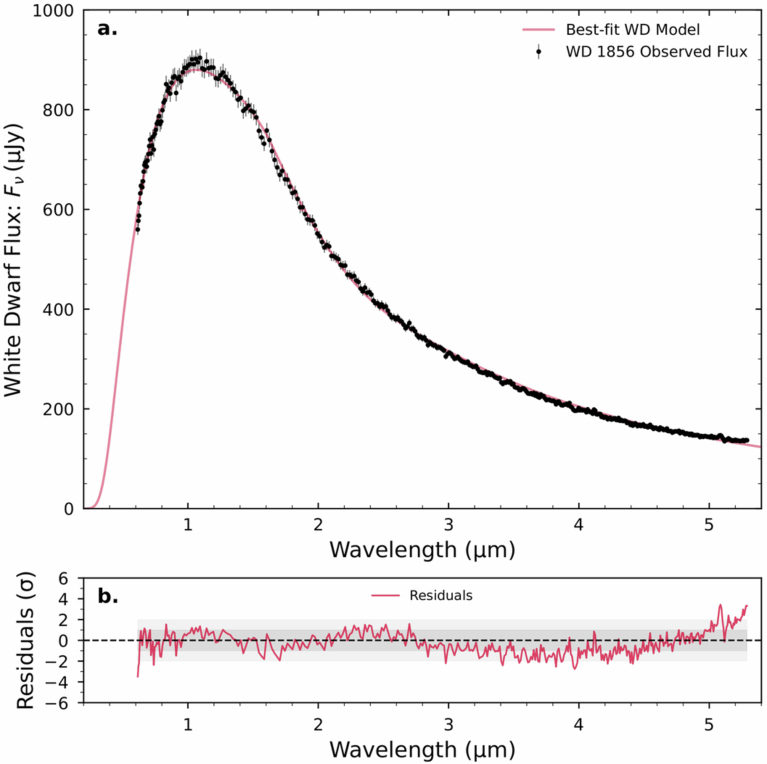
WD 1856 host white dwarf spectrum. **a**, JWST NIRSpec PRISM out-of-transit spectrum of WD 1856 b (black) compared with a best-fitting white dwarf model (red). The 1*σ* error bars include a minimum 2% absolute flux calibration uncertainty. **b**, Residuals between the data and the best-fitting model. Source data

We extracted a calibrated out-of-transit NIRSpec PRISM stellar spectrum for WD 1856 using the FIREFLy data reduction. Starting from the cleaned 2D images, we further flat-fielded, flux-calibrated and extracted the resulting host spectrum. The resulting stellar spectra are shown in Extended Data Fig. 3.

We determined the atmospheric parameters of the host white dwarf by fitting the out-of-transit system flux. We minimized the *χ*^2^ for a model suitable for cool white dwarfs^14^ defined by three parameters: the effective temperature of the white dwarf, *T*_eff_, its photospheric hydrogen-to-helium abundance ratio and the solid angle πR_*_^2^/*D*^2^. Because the distance *D* is known from the Gaia DR3 parallax, the solid angle directly constrains the radius of the white dwarf. The radius, in turn, determines the mass and surface gravity of the white dwarf given theoretical white dwarf structure models^37^. The best-fit solution (Extended Data Fig. 3) corresponds to *T*_eff_ = 4,920 K, log*g* = 8.05 and *N*_H_/*N*_He_ = 4.1. This solution yields an H*α* line that extends 2% below the continuum, which is consistent with previously obtained optical spectroscopy^11^ (not considered here in our fit). We also attempted to fit the PRISM spectrum using pure-hydrogen models (that is, without considering *N*_H_/*N*_He_ as a free parameter), but the best-fit solution yields a much worse fit to the PRISM data than the mixed H and He atmosphere solution. We calculated limb-darkening coefficients for the best-fitting white dwarf model (using the approach from ref. ^38^), which we then fixed during the WD 1856 b spectroscopic light curve fits for our two data reductions.

### Transmission spectrum modelling

The grazing transit geometry of WD 1856 b, coupled with the clear presence of planetary nightside thermal emission, requires a new modelling approach. The transmission spectrum for a planet with a grazing transit and nightside thermal emission can be written as^39^:

7 $${\varDelta }_{\lambda }=\frac{{A}_{{\rm{p}}({\rm{t}}{\rm{o}}{\rm{p}})}-{\int }_{{A}_{{\rm{p}}}}{{\mathcal{T}}}_{\lambda }{\rm{d}}A}{{\rm{\pi }}{R}_{\ast }^{2}}\left(\frac{1}{1+\frac{{F}_{{\rm{p}}({\rm{n}}{\rm{i}}{\rm{g}}{\rm{h}}{\rm{t}}),\lambda }}{{F}_{\ast ,\lambda }}}\right)$$

in which *A*_p(top)_ is the area of the planet overlapping the star at the top of the modelled atmosphere (given by equation (1) with *R*_p_ = *R*_p,top_), ${{\mathcal{T}}}_{\lambda }$ is the atmospheric transmissivity (${{\rm{e}}}^{-{\tau }_{\lambda }}$, in which *τ*_*λ*_ is the slant optical depth) in the area element d*A* and *F*_p(night)__,*λ*_ and F_*,*λ*_ are the observed fluxes from the planetary nightside and white dwarf at Earth, respectively. We reduce the area integral in the first term to a single integral over the fractional annuli of the planet overlapping the star (that is, d*A* = *A*_p_(*r*_*i*,up_) − *A*_p_(*r*_*i*,low_), in which we use the radii of the upper and lower boundaries of each atmospheric layer in place of *R*_p_ in equation (1)). The first term in equation (7) represents the wavelength-dependent effective area of the fraction of the planet overlapping the white dwarf, relative to the projected disk area of the white dwarf. The second term accounts for the ‘nightside pollution’/dilution of the transit depth^17^ owing to thermal emission from the planetary hemisphere facing the observer.

Transmission spectra of WD 1856 b can also be expressed in terms of emergent fluxes by using the solid angle relation between the observed flux and the emergent (surface) flux, such that equation (7) becomes:

8 $${\varDelta }_{\lambda }=\frac{{A}_{{\rm{p}}({\rm{t}}{\rm{o}}{\rm{p}})}-{\int }_{{A}_{{\rm{p}}}}{{\mathcal{T}}}_{\lambda }{\rm{d}}A}{{\rm{\pi }}{R}_{\ast }^{2}}\times \left(\frac{1}{1+\frac{{R}_{{\rm{p}},({\rm{n}}{\rm{i}}{\rm{g}}{\rm{h}}{\rm{t}}),\lambda }^{2}}{{R}_{\ast }^{2}}\frac{{F}_{{\rm{p}}({\rm{n}}{\rm{i}}{\rm{g}}{\rm{h}}{\rm{t}}),{\rm{s}}{\rm{u}}{\rm{r}}{\rm{f}},\lambda }}{{F}_{\ast ,{\rm{s}}{\rm{u}}{\rm{r}}{\rm{f}},\lambda }}}\right)$$

in which *R*_p,(night),*λ*_ is the radius of the emitting thermal photosphere on the nightside (nominally the *τ*_*v*,*λ*_ = 2/3 pressure level, in which *τ*_*v*,*λ*_ is the vertical optical depth integrated downwards from the top of the atmosphere). The planet–star surface flux ratio featured in equation (8) is a standard output from radiative transfer codes used to calculate exoplanet emission spectra. Similarly, the transmissivity, ${{\mathcal{T}}}_{\lambda }$, is already calculated by radiative transfer codes calculating standard transmission spectra. Therefore, to calculate the transmission spectra of WD 1856 b, we can construct a model atmosphere and then calculate both the transmissivity from the slant optical depth and the emergent planet–host flux ratio. The observed transmission spectrum then represents a product between a grazing transit transmission spectrum and an ‘upside-down’ emission spectrum.

### Atmospheric retrieval analysis

We infer the atmospheric properties of WD 1856 b using the open-source Bayesian atmospheric retrieval code POSEIDON^15,16^. We model the atmosphere of WD 1856 b using 100 layers spaced uniformly in log-pressure from 10^−7^ to 100 bar. We assume that the atmosphere is well mixed, with consistent atmospheric properties at the day–night terminator and at the nightside, such that only a single set of parameters describe the atmospheric state. We fit for the planetary radius at the 10 bar pressure level and the planetary mass, while fixing the white dwarf radius to R_*_ = 0.0131 *R*_Sun_ and the transit impact parameter to 7.430234 (as the impact parameter uncertainty is already marginalized into the *R*_p_/R_*_ uncertainties, it does not need to be an independent free parameter). We include the log_10_ mixing ratios of the following molecules as free parameters: CH_4_, NH_3_, H_2_O, CO_2_, CO, HCN, C_2_H_2_, C_2_H_4_, C_2_H_6_, H_2_S and PH_3_. The remainder of the atmosphere is composed of H_2_ and He with an abundance ratio of He/H_2_ = 0.17, consistent with the giant planets in the Solar System^40^. We parameterize the temperature profile of WD 1856 b using an adaptation of a prescription used for brown dwarfs^41^. This prescription retrieves the temperature at nine pressure nodes (spaced uniformly per decade in pressure from 10^−^^6^ to 100 bar) and interpolates between them with a spline. The nine free parameters defining this temperature profile are the 100 mbar temperature and eight Δ*T*_*i*_ parameters encoding the temperature difference between each pair of nodes. Given the low external irradiation of WD 1856 b, we restrict Δ*T*_*i*_ > 0 to consider physically plausible profiles with temperature monotonically increasing with pressure. Finally, we fit for a three-parameter aerosol model consisting of a power law scattering slope (with exponent *γ*) and an optically thick cloud-top pressure^15^. We do not consider inhomogenous clouds around the terminator, as only a small fraction of the terminator of WD 1856 b occults the surface of the white dwarf during transit.

Our retrieval model is thus defined by 25 free parameters, which we fit using MultiNest’s^42–44^ Python wrapper PyMultiNest^45^ with 1,000 live points. The priors for each parameter are summarized in Extended Data Table 2. We calculate Bayes factors (that is, odds ratios; ${\mathcal{B}}$) using Bayesian model comparisons between nested retrieval models, with the retrieval model statistics summarized in Extended Data Table 3. For consistency with the exoplanet literature, we also convert the Bayes factor between two nested models (for example, our reference model and a model excluding CH_4_) into an ‘equivalent detection significance’, *Nσ*, using a standard relation^46^. We note, however, that there are several caveats associated with the Bayes factor to detection significance mapping, so our preferred statistic for model preference is the Bayes factor/odds ratio (see ref. ^47^).

We calculate model transmission spectra of WD 1856 b by solving equation (8) on a wavelength grid ranging from 0.5 to 5.6 μm at *R* = 20,000. We sample high-resolution pre-computed cross-sections^39^ onto this wavelength grid, using the following line list sources: CH_4_ (ref. ^48^), NH_3_ (ref. ^49^), H_2_O (ref. ^50^), CO_2_ (ref. ^51^), CO (ref. ^52^), HCN (ref. ^53^), C_2_H_2_ (ref. ^54^), C_2_H_4_ (ref. ^55^), C_2_H_6_ (ref. ^55^), H_2_S (ref. ^56^) and PH_3_ (ref. ^57^). We also include continuum opacity from H_2_ and He collision-induced absorption^58^ and Rayleigh scattering. For the host flux, we use the best-fit white dwarf model shown in Extended Data Fig. 3. Our model transmission spectra are finally convolved with the NIRSpec PRISM point spread function and binned down to the resolution of the observations to calculate the likelihood of each location in the retrieval model parameter space.

**Extended Data Fig. 4:**
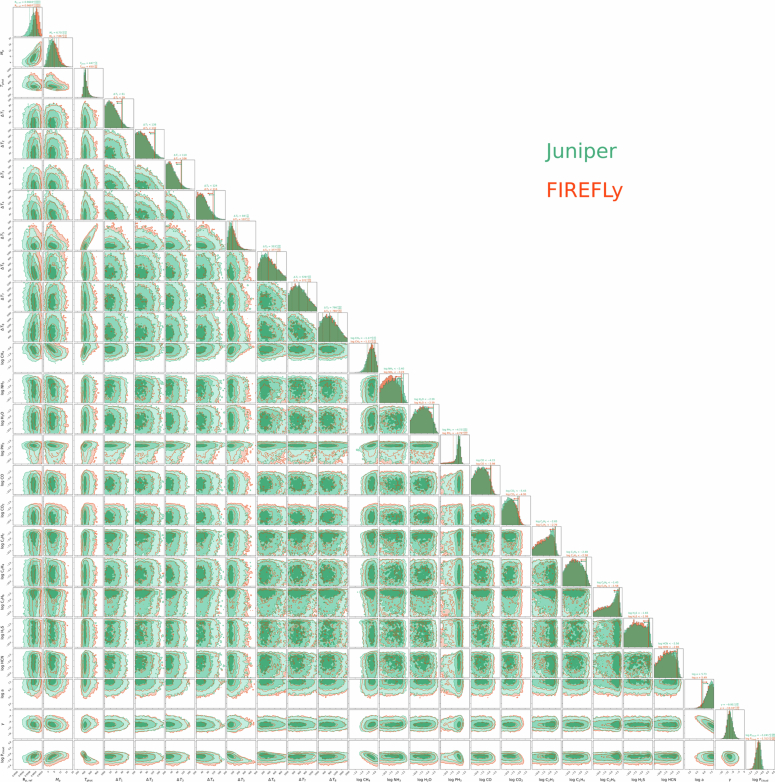
Full posterior distributions for the FIREFLy (orange) and Juniper (green) data reductions. The histograms show the marginalized posterior probability density for each model parameter for each data reduction, with the median (solid lines) and 1*σ* credible interval (dashed lines) overplotted for well-constrained parameters. Parameters without clear upper and lower limits, such as non-detected molecules, have 2*σ* annotated instead (arrows). The 2D correlation panels are shaded according to the 1*σ* (darkest) through to the 3*σ* credible region.

Although Fig. 3 compares several retrieved atmospheric properties between the FIREFLy and Juniper data reductions, we provide the full posterior distributions in Extended Data Fig. 4. We find excellent agreement between FIREFLy and Juniper for all retrieved parameters.

**Extended Data Fig. 5:**
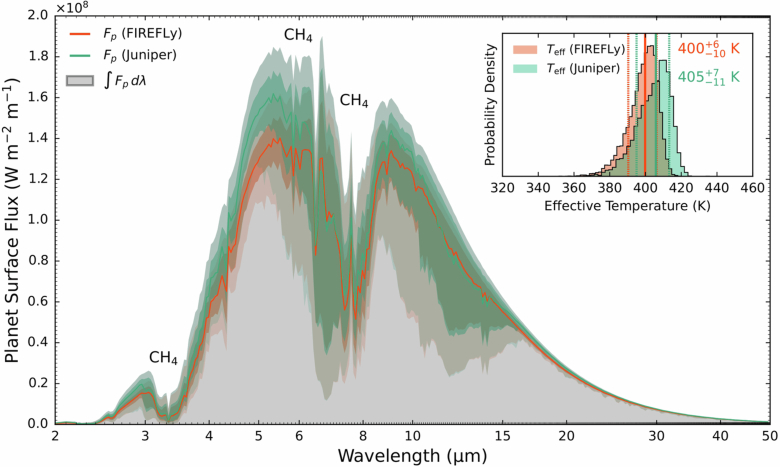
Retrieved emergent flux and effective temperature. **a**, The retrieved emergent surface flux of WD 1856 b for the FIREFLy (orange curve and credible interval shading) and Juniper (green curve and credible interval shading) reductions are shown extrapolated out to 50 μm. The integral under the curve (grey shading) is used to determine the effective temperature, *T*_eff_, corresponding to the surface flux of each model atmosphere. **b**, Posterior distributions of the effective temperature of WD 1856 b for the FIREFLy and Juniper datasets, with the 1*σ* credible region annotated.

To interpret the thermal history of WD 1856 b, we also calculate posterior distributions for the planetary effective temperature from our retrieval results. We calculated the emergent planetary surface flux of WD 1856 b for each atmosphere in the full set of posterior samples from both the FIREFLy and Juniper reductions on a wavelength grid from 1 to 50 μm. For each set of atmospheric parameters, we calculate the corresponding effective temperature using the Stefan–Boltzmann law: ${T}_{{\rm{eff}}}={\left(\frac{1}{{\sigma }_{{\rm{SB}}}}\int {F}_{{\rm{p}},{\rm{surf}},\lambda }{\rm{d}}\lambda \right)}^{1/4}$. Extended Data Fig. 5 shows our retrieved surface flux spectrum for WD 1856 b for both data reductions and the corresponding *T*_eff_ posterior distributions. Using the lowest 1*σ* credible interval (from FIREFLy) and the highest 1*σ* credible interval (from Juniper), we find a range of 390–412 K for *T*_eff_. We similarly report the 1*σ* range encompassing both data reductions for *M*_p_ in the main text. The roughly 10 K 1*σ* uncertainty on *T*_eff_ is driven by the numerous CH_4_ bands detected in our NIRSpec PRISM data setting the relative amplitude of other CH_4_ features at longer wavelengths. However, the potential presence of other hydrocarbons, such as C_2_H_6_, allows a larger surface flux uncertainty in which these species absorb in the mid-infrared (for example, 10–15 μm), which increases the uncertainty in the integrated power and hence *T*_eff_. Longer wavelength observations of WD 1856 b with MIRI LRS/MRS, such as those planned in JWST Cycle 4 (GO-9033 and GO-9157), will constrain *T*_eff_ even further.

#### Mie scattering retrievals

We have established that models including aerosol opacity are required to explain the transmission spectrum of WD 1856 b. Specifically, our free retrieval analysis above infers an opaque cloud deck near 100 mbar and a haze to explain the power-law scattering slope shortwards of 1 μm. The enhanced scattering slope indicates a collection of small particles in the upper atmosphere, but our parametric description is agnostic to the specific aerosol composition. Here we consider retrievals including Mie scattering to investigate which specific aerosol species are consistent with the transmission spectrum of WD 1856 b.

The composition of small, Mie scattering particles can be potentially identified by means of aerosol absorption features at infrared wavelengths, whereas their particle size is encoded by the scattering slope. We assess here which aerosol species and particle sizes can explain the observed scattering slope by means of retrievals including compositionally specific Mie scattering aerosols. We do not test directly for specific species causing the opaque cloud deck, as this deck is probably composed of large particles with muted resonance features^59,60^. Because such a condensate cloud deck has no spectroscopic features, it is not possible to determine the composition unless condensates are lofted above the deck and become smaller in size.

We use the Mie scattering retrieval module and database introduced in POSEIDON v1.2 (ref. ^61^). Our Mie scattering retrievals use aerosol extinction cross-sections pre-computed from refractive indices. We mainly consider a simple aerosol model parameterized by the log_10_ mean particle size (log*r*_m_ ${\mathcal{U}}\,[-3,1]$) and aerosol log_10_ volume mixing ratio (log aerosol ${\mathcal{U}}\,[-30,-1]$)—representing a well-mixed aerosol uniformly distributed within the atmosphere. We also tested more complex aerosol models that fit for pressure-dependent aerosol mixing ratios but these all reduced to a pressure-independent model. Our Mie retrievals use a six-parameter pressure–temperature (*P*–*T*) profile^62^. We conduct these Mie retrievals on the FIREFLy data reduction.

We ran retrievals with a suite of aerosol species representing three different aerosol formation regimes that could be relevant in upper atmosphere of WD 1856 b. The first aerosol regime represents disequilibrium hazes and soot species that can be produced by photochemistry: Titan tholins (tholins^63,64^), carbon soot (C (ref. ^65^)), water-rich organic haze at two temperatures (ExoHaze 300K, ExoHaze 400K (ref. ^66^)) and hexene (C_6_H_12_ (ref. ^67^)). The second aerosol regime represents the myriad of sulfide and chloride clouds that form in brown dwarfs at the T–Y transition (400–1,300 K)^27^ alongside Cr: chromium (Cr; Lynch and Hunter in ref. ^68^), magnesium sulfide (MnS (ref. ^69^)), sodium sulfide (Na_2_S (ref. ^27^)), zinc sulfide (ZnS (ref. ^70^)) and potassium chloride (KCL; Palik and Addamiano in ref. ^71^) (ordered by condensation temperature). The third aerosol regime consists of condensed ices that form deep cloud decks in Solar System planets and potentially cooler Y dwarfs (≤400 K)^72,73^. These ices could cause the opaque cloud deck found in our retrievals above, which are then lofted to higher atmospheric pressures to cause the observed scattering slope, or they could condense in situ in the colder upper atmosphere: water ice (H_2_O (ref. ^74^)), ammonia ice (NH_3_ (ref. ^75^)) and methane ice (CH_4_ (ref. ^76^)) (ordered by condensation temperature).

We find that all aerosol species, with the exception of MnS and hexene, provide good fits to the scattering slope and only imprint weak absorption features into the transmission spectrum. Using Bayesian model comparisons, the best-fit haze and soot species is the water-rich organic ExoHaze (the 400K variant), the best-fit T–Y dwarf cloud species is KCl and the best-fit ice is NH_3_. Of these three aerosols, KCl has the highest Bayesian evidence. The potential presence of KCl would be consistent with expectations for cold T–Y dwarf models^27^, in which KCl forms the highest, low-density cloud. However, we note that a simple grey cloud deck + haze model (as used in the main text) is preferred over KCl by about 2*σ*. Therefore, the present data for WD 1856 b is not sufficiently precise to identify a clear preference for which specific aerosols are present in the atmosphere of WD 1856 b.

**Extended Data Fig. 6:**
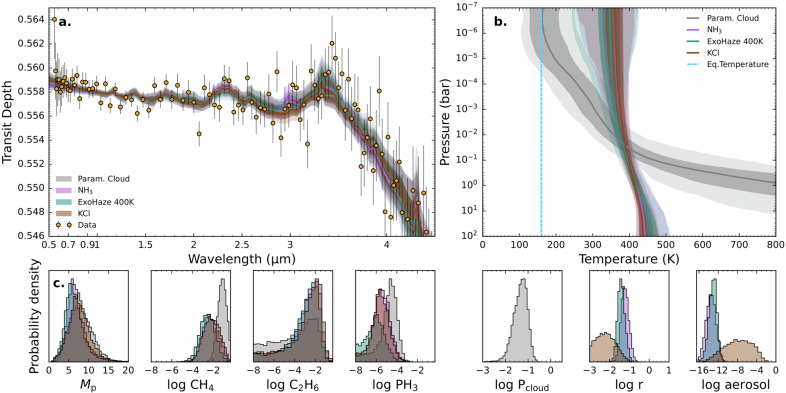
Composition-specific Mie scattering retrievals. **a**, Three retrieval models including Mie scattering (NH_3_ ice, purple; ExoHaze 400K, green; and KCl, orange) are compared with our reference non-Mie scattering grey cloud deck + haze model (grey). All four retrieval models produce similar fits to the transmission spectrum of WD 1856 b (FIREFLy; orange circles with 1*σ* error bars). **b**, Corresponding *P*–*T* profiles. The Mie scattering profiles are colder in the deeper atmosphere owing to the inability of these models to produce an optically thick deep cloud deck. **c**, Corresponding posterior distributions for key atmospheric properties.

Our Mie scattering retrievals provide insights into the range of particle sizes and abundances compatible with the short wavelength scattering slope WD 1856 b (Extended Data Fig. 6). The ExoHaze and NH_3_ ice models favour a collection of small particles (about 0.03 μm) with low mixing ratios (about 10^−14^), whereas the KCl model favours even smaller particles (roughly 0.01 μm) with a higher abundance (about 10^−8^). Compared with our default grey cloud deck + haze retrieval model, we find consistent results for other model parameters to within 1*σ*. In particular, we show that the retrieved planetary mass is not sensitive to the assumed aerosol model. We do find roughly 1 dex lower median CH_4_ abundances for the Mie scattering retrievals and hence a lower C/H ratio, but the CH_4_ abundance distribution is still consistent with our results in the main text. We note that the marginal evidence of C_2_H_6_ strengthens when including Mie scattering compared with the deck + haze model (Extended Data Fig. 6) but this molecule is not strongly detected with the present data.

Our retrieved temperature structure from the Mie scattering retrievals also indicates an atmosphere that is much warmer than the equilibrium temperature of WD 1856 b (Extended Data Fig. 6). As with our grey cloud and haze retrieval, we also find a temperature of about 400 K in the thermal photosphere near 10–100 mbar. However, because the Mie scattering retrievals cannot produce an optically thick cloud deck at the pressures required to obscure thermal emission from the deep atmosphere (approximately 10^−1.5^ bar), the Mie retrievals compensate by making the *P*–*T* profile essentially isothermal in the deep atmosphere (that is, the retrieved *P*–*T* profile shown in Fig. 2 is more physical). We note that our uniform aerosol Mie scattering retrievals are incompatible with the *P*–*T* profile used in the main text^41^, as a collection of small aerosols are not able to simultaneously block the deep adiabatic thermal flux and fit the scattering slope. The *P*–*T* profile parameterization chosen here^62^ tends to favour a nearly isothermal upper atmosphere, which suffices for the exploration of the aerosol properties consistent with the scattering slope of WD 1856 b. Future explorations of the cloud structure and radiative properties of WD 1856 b, such as composite cloud models with multiple scattering, are a rich area to deepen our understanding of the atmosphere of WD 1856 b.

### Evolution of the WD 1856 system

#### Host progenitor and white dwarf

We examined the evolution of the progenitor star of WD 1856 by consulting the MIST evolutionary models^77^ for non-rotating solar-metallicity stars in the appropriate mass range (${M}_{{\rm{progenitor}}}=1.3{6}_{-0.18}^{+0.29}\,{M}_{{\rm{Sun}}}$). From these models, we extracted fiducial estimates of the main sequence lifetime (${4}_{-1.8}^{+2.4}\,{\rm{Gyr}}$) using an initial–final mass relation^78,79^, the duration of the thermally pulsing AGB stage ($1.5{5}_{-0.10}^{+0.26}\,{\rm{Myr}}$) and the post-AGB/pre-white-dwarf stage ($0.03{4}_{-0.002}^{+0.053}\,{\rm{Myr}}$). The latter is defined here as the elapsed time between the final thermal pulse and the cooling of the exposed core to an effective temperature of 100,000 K. This yields a total system age of $9.{4}_{-1.9}^{+2.5}\,{\rm{Gyr}}$.

We calculated the cooling age of the white dwarf host by evolving MESA white dwarf models of the appropriate mass down to *T*_eff_ = 4,920 K. We used MESA r23.05.1 (ref. ^80^). This MESA release now includes carbon–oxygen fractionation^81^, which is important here as the white dwarf is in the process of crystallizing. A standard helium layer of $\log {M}_{{\rm{He}}}/{M}_{* }=-2$ was assumed, whereas a relatively thin hydrogen layer of $\log {M}_{{\rm{H}}}/{M}_{* }=-6$ was used. This is much thinner than the canonical value of $\log {M}_{{\rm{H}}}/{M}_{* }=-4$ (ref. ^82^) but is motivated by the fact that the model atmosphere analysis points to an atmosphere containing a mix of hydrogen and helium. This presumably requires the superficial convection zone to extend just below the hydrogen layer, thereby diluting hydrogen with helium. From this constraint, we can estimate $\log {M}_{{\rm{H}}}/{M}_{* }=-6$ (ref. ^83^). Cooling calculations were performed for different carbon–oxygen core composition profiles to account for current model uncertainties: a standard profile predicted by stellar evolution^80^ and an asteroseismologically derived stratification^84^ were used. We also calculated cooling models using different electron thermal conductivities^85,86^ to account for current uncertainties at the transition between the regimes of moderate and strong degeneracy^87^. From this analysis, we find a cooling age of 5.4 ± 0.7 Gyr, in which the uncertainty includes the systematic uncertainty sources listed above and a 2% uncertainty on the *T*_eff_ of the star and mass typical of white dwarfs in this temperature range^88^. This cooling age is consistent with estimates produced by other stellar evolution codes^82,89^.

#### Thermal history of WD 1856 b

We reconstructed the thermal evolution of WD 1856 b under the assumption that the cooling of the planet after migration has been similar to the cooling undergone by a substellar object after formation. We used cooling models from the ATMO2020 (ref. ^90^) and Sonora Bobcat^91^ model grids. Each grid tabulates global quantities such as luminosity, effective temperature, radius and surface gravity as a function of age for substellar objects of a given mass and bulk chemical composition, starting from an initial condition with high entropy. Both provide self-consistent evolutionary–atmospheric modelling frameworks, in which the structure and evolution of the fully convective, adiabatic interior are computed with a cloudless, non-grey, rainout-chemical-equilibrium atmosphere as the surface boundary condition. The most important difference between ATMO2020 and Sonora Bobcat is that the former neglects some relevant opacity sources at effective temperatures above 2,000 K, leading to faster cooling at high temperatures in the ATMO2020 models. ATMO2020 provides models of solar-metallicity objects, whereas Sonora Bobcat provides models for both solar-metallicity and metal-enriched ([M/H] = +0.5) objects. We consider these three sets of models in our analysis below.

Reconstructing the thermal history of WD 1856 b requires us to choose a model grid and specify three parameters: planetary mass (*M*_p_), current planetary effective temperature (*T*_eff,p_) and current white dwarf cooling age (*t*_wd_). For a given *M*_p_ and model grid, we obtained the effective temperature as a function of time by adding a uniform offset (*t*_0_) to the model age (*t*_p_) such that the model temperature matches *T*_eff,p_ at *t*_p_ = *t*_wd_ − *t*_0_ (using linear interpolation between the tabulated model ages and temperatures). The cooling models predict a high effective temperature (about 1,500–3,000 K) at *t*_0_; these values are plausible for planets that have been tidally heated during high-eccentricity migration^92,93^ or have survived a common-envelope phase^8,9,94^. We therefore interpret *t*_0_ as an estimate of the time of the planet’s reheating during migration, expressed as a white dwarf cooling age. Because cooling is rapid at high *T*_eff,p_, our estimate of *t*_0_ is robust to theoretical uncertainties in what temperature the planet should be immediately after migration.

We considered cooling models with *M*_p_ between 0.5 and 20 *M*_J_, covering the range of samples from the atmospheric retrieval posterior distributions for the FIREFLy and Juniper JWST data reductions. Each grid samples a finite number of mass values; when considering objects of arbitrary mass between grid points, we used the cooling model with the nearest mass on the grid. Both the ATMO2020 and Sonora Bobcat models are spaced by about 0.5–1.0 *M*_J_ in mass over the range we consider, so our approach does not introduce substantial error in a given reconstruction compared with interpolating between adjacent models.

We generated ensembles of possible thermal histories using the mass and effective temperature constraints derived from the NIRSpec PRISM transmission spectrum of WD 1856 b. Specifically, we considered nearly 10,000 values of *M*_p_ from the atmospheric retrieval posterior distribution alongside the nearly 10,000 corresponding values of *T*_eff,p_ obtained using the procedure described above for each data reduction (see the ‘Atmospheric retrieval analysis’ section). Our samples of *M*_p_ and *T*_eff,p_ are not statistically independent, as each pair of values is derived from a single sample from the distribution of atmospheric models consistent with our NIRSpec PRISM transmission spectrum. This has an important influence on the range of thermal histories that we can infer from the data, because the cooling rate is a sensitive function of mass.

**Extended Data Fig. 7:**
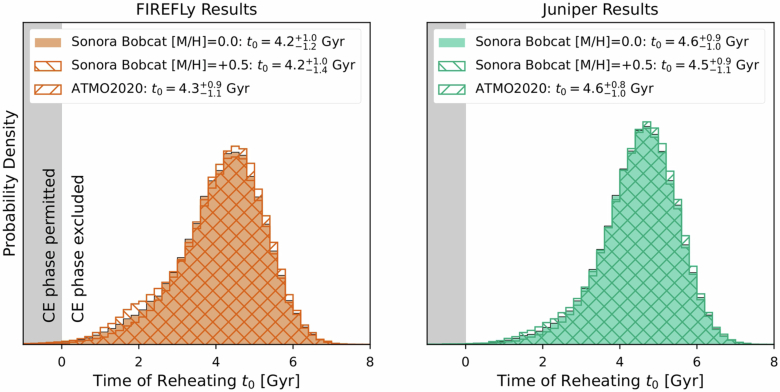
The inferred time of reheating of WD 1856 b using different cooling models. Each histogram contains about 100,000 possible histories generated from the same set of properties ((*M*_p_, *T*_eff,p_, *t*_wd_), the first two from the atmospheric retrieval samples) but different cooling models (shading and hatching). The left panel corresponds to the FIREFLy reduction and the right is for Juniper. The legend summarizes the 1*σ* credible region of *t*_0_ for each cooling model. Only values of *t*_0_ < 0 (shaded regions) allow for a common-envelope evolutionary phase.

On the other hand, our estimated *t*_wd_ is independent of our atmospheric retrieval analysis. For each pair of *M*_p_ and *T*_eff,p_ values, we generated ten random values of *t*_wd_ drawn from a Gaussian distribution with mean 5.4 Gyr and standard deviation 0.7 Gyr. Each ensemble therefore comprises about 100,000 possible thermal histories consistent with the transmission spectrum of WD 1856 b. We generated one ensemble for each model grid. Extended Data Fig. 7 shows the distribution of calculated *t*_0_ values for the three cooling models and two data reductions, from which we derive a statistical constraint on *t*_0_. The results reported in the main text were obtained using the solar-metallicity Sonora Bobcat models (solid orange and green histograms; see also Fig. 4). If we use the ATMO2020 models, we find a comparable ${t}_{0}=4.{3}_{-1.1}^{+0.9}\,{\rm{Gyr}}$ for FIREFLy and $4.{6}_{-1.0}^{+0.8}\,{\rm{Gyr}}$ for Juniper. Using the metal-enriched Sonora Bobcat models yields ${t}_{0}=4.{2}_{-1.4}^{+1.0}\,{\rm{Gyr}}$ for FIREFLy and $4.{5}_{-1.1}^{+0.9}\,{\rm{Gyr}}$ for Juniper. The conclusions we draw from modelling the thermal evolution of WD 1856 b are therefore robust to both the JWST data reduction and the choice of cooling models, given the models available at present.

In a small fraction of cases (<0.15%) for each ensemble, we calculate values *t*_0_ < 0. These correspond to the highest *M*_p_ values sampled from the atmospheric retrieval posterior. Negative values of *t*_0_ arise in these cases because we have calculated *t*_0_ by extrapolating the cooling models back to the effective temperatures expected among newborn brown dwarfs of about 20 *M*_J_ (>2,000 K). However, these results are unphysical according to the interpretation of *t*_0_ as the time elapsed between the end of the AGB phase and the reheating/migration of the planet. If we stipulate that reconstructed thermal histories be truncated for *t*_0_ < 0, then these few cases are consistent with common-envelope evolution in that it is possible for the planet to have achieved its current temperature by passively cooling since the end of the AGB phase (albeit from a cooler, lower-entropy state than those implied in cases with *t*_0_ > 0). The fact remains, however, that most of the cases (>99.85%) imply *t*_0_ values that cannot coincide with a common-envelope phase in all three ensembles. Thus, we conclude that reheating during the white dwarf phase (consistent with high-eccentricity migration) is preferred over reheating during common-envelope evolution at >2*σ* (for FIREFLy) and >3*σ* (for Juniper). Further theoretical study is needed to corroborate or qualify this conclusion, as we describe below.

**Extended Data Fig. 8:**
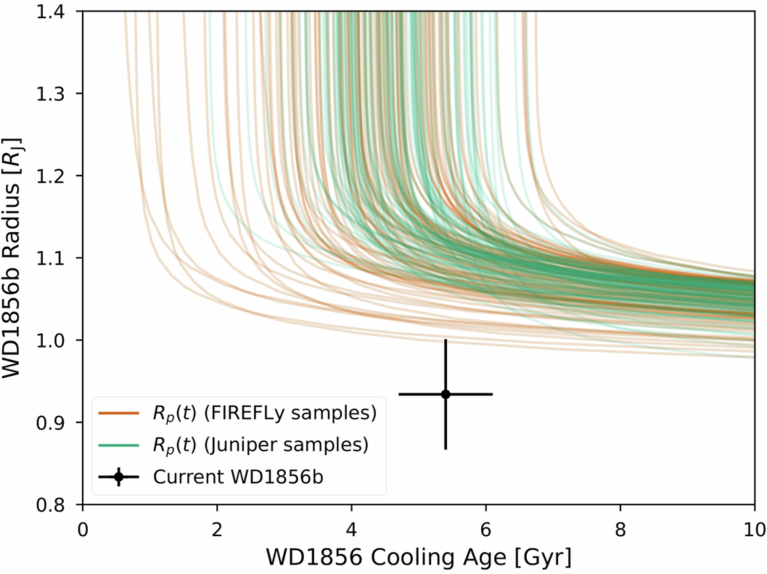
Radius evolution of WD 1856 b. The observed radius of WD 1856 b and white dwarf cooling age (black point and 1*σ* error bars) and compared with the modelled radius as a function of its age for FIREFLy (orange curves) and Juniper (green curves). The models arise from the temperature-based reconstruction of the thermal evolution of WD 1856 b.

Our method of reconstructing thermal histories is based on backward extrapolation of the effective temperature only. However, the cooling models also predict the evolution of the radius of WD 1856 b; these predictions should agree in principle. In Extended Data Fig. 8, we show the radius evolution implied by our reconstruction method for 100 samples from the Sonora Bobcat ensemble for both data reductions. For the observed radius, we use the best-fitting value *R*_p_ = 0.911 ± 0.020 *R*_J_ from the FIREFLy reduction. We also include a systematic error of ±0.050 *R*_J_ given by the range of best-fitting radius values covered by the two data reductions, for a total uncertainty of ±0.054 *R*_J_. We see that many of the temperature-based reconstructions overestimate the radius of WD 1856 b by about 2*σ*. Future efforts to understand the thermal evolution of WD 1856 b should reproduce both the effective temperature and radius. A clue as to the origin of this discrepancy comes from the heavy-element enrichment of the envelope of WD 1856 b, suggested by our retrieved CH_4_ abundance, as planetary radius decreases with increasing metallicity at a fixed mass and internal entropy. Model grids of comparable quality with ATMO2020 and Sonora Bobcat that are applicable to objects as massive (about 7 *M*_J_) and metal-rich (about 100 × solar) as WD 1856 b have not been developed or published to our knowledge.

We note that we neglected the irradiation of WD 1856 b by the host white dwarf in our reconstruction of the planet’s thermal history. Irradiation is a key ingredient in modelling the structure and evolution of short-period exoplanets around main-sequence stars, such as hot Jupiters^95,96^. The importance of irradiation in the case of WD 1856 b can be gauged by calculating the ratio of the power emitted from the photosphere of the planet to the power incident on the planet from the star:

9 $${\mathcal{R}}=4{\left(\frac{{T}_{{\rm{eff}},{\rm{p}}}}{{T}_{{\rm{eff}},\ast }}\right)}^{4}{\left(\frac{{a}_{{\rm{orb}}}}{{R}_{\ast }}\right)}^{2},$$

in which *T*_eff,*_ and *T*_eff,p_ are measured effective temperatures of the host star and planet, respectively, R_*_ is the host radius and *a*_orb_ is the orbital semimajor axis (assuming a near-circular orbit). Using the system parameters as determined in this work, we calculate ${\mathcal{R}}\approx 25$, indicating that the self-luminosity of the planet overwhelms the power received from the star. Our reconstructed histories generally find that this ratio was larger in the past (except perhaps in the first several Myr after the white dwarf formed). Thus, we argue that irradiation has had a small effect on the previous thermal evolution of WD 1856 b. It would be of interest to self-consistently model the evolution of a substellar body with time-dependent irradiation, as would be the case in proximity to a cooling white dwarf. We leave this for future work.

#### Alternatives to reheating during migration

We considered several alternative explanations for the increased effective temperature of WD 1856 b, all of which we deemed implausible or unlikely. We briefly describe each of them here, along with our reasoning.

First, the observed effective temperature of WD 1856 b cannot be explained purely by passive cooling over the system’s total age of about 10 Gyr (ref. ^1^). This is readily ruled out by consulting theoretical cooling models^90,91^. To have an effective temperature of about 400 K at an age of 10 Gyr, WD 1856 b would need to have a mass of approximately 24 *M*_J_. Our observations rule out such a high mass at >3*σ* confidence.

The mass of WD 1856 b may be above the threshold for deuterium fusion in its core (about 13 *M*_J_) within 2*σ*. However, although it is possible that WD 1856 b was once heated internally by nuclear reactions, this cannot explain its present-day properties. Models of deuterium-burning brown dwarfs predict a total luminosity many orders of magnitude greater than that of WD 1856 b (ref. ^97^). The duration of deuterium burning (about 3–50 Myr depending on mass^97^) is much shorter than the total age of the system, so the primordial deuterium WD 1856 b would have been destroyed early in the main-sequence lifetime of the host.

Owing to the proximity of WD 1856 b to its host, tidal interactions are another possible heat source inside the planet; this would be analogous to the heating of the Galilean satellite Io through its tidal interaction with Jupiter^98^. For tidal heating to operate, the orbit of WD 1856 b would need to be slightly eccentric rather than circular as is typically assumed. Assuming that the power dissipated by tidal friction is equal to the total power emitted by WD 1856 b, we calculate the effective temperature of the planet, using the standard ‘equilibrium tide’ theory^99^, as:

10 $$\begin{array}{c}{T}_{{\rm{e}}{\rm{f}}{\rm{f}},{\rm{p}}}={\left(\frac{21}{8{\rm{\pi }}}\frac{{G}^{2}{M}_{\ast }^{3}{R}_{{\rm{p}}}^{3}}{{\sigma }_{{\rm{S}}{\rm{B}}}{a}_{{\rm{o}}{\rm{r}}{\rm{b}}}^{9}}{k}_{2{\rm{p}}}{\tau }_{{\rm{p}}}{e}_{{\rm{o}}{\rm{r}}{\rm{b}}}^{2}\right)}^{1/4}\\ \,\approx \,400\,{\rm{K}}{\left(\frac{{M}_{\ast }}{0.60{M}_{\odot }}\frac{{R}_{{\rm{p}}}}{0.91{R}_{{\rm{J}}}}\right)}^{3/4}{\left(\frac{{a}_{{\rm{o}}{\rm{r}}{\rm{b}}}}{0.02{\rm{A}}{\rm{U}}}\right)}^{-9/4}\\ \,\times \,{\left(\frac{{k}_{2{\rm{p}}}}{0.4}\frac{{\tau }_{{\rm{p}}}}{0.1{\rm{s}}}\right)}^{1/4}{\left(\frac{{e}_{{\rm{o}}{\rm{r}}{\rm{b}}}}{0.02}\right)}^{1/2}.\end{array}$$

Here *G* is the gravitational constant, *σ*_SB_ is the Stefan–Boltzmann constant, M_*_ is the mass of the host white dwarf, *R*_p_ is the radius of WD 1856 b and *a*_orb_ and *e*_orb_ are respectively the orbital semimajor axis and eccentricity (with *e*_orb_ ≪ 1). The quantities *k*_2p_ and *τ*_p_, known respectively as the tidal Love number and tidal lag time, characterize the dissipation inside WD 1856 b in the standard equilibrium tidal theory. The reference values of M_*_, *R*_p_ and *a*_orb_ used in equation (10) match the observed system parameters. For *k*_2p_ and *τ*_p_, we use values similar to those inferred for Jupiter’s dissipation of the tide raised by Io^100,101^. We see that tidal heating could, in principle, sustain the observed effective temperature of WD 1856 b for an orbital eccentricity of about 0.02 (the highly uncertain values of *k*_2p_ and *τ*_p_ notwithstanding). This would be consistent with orbital circularization in the end stage of high-eccentricity migration. However, the same dissipation would damp the orbital eccentricity on a characteristic timescale of roughly 0.075 Gyr (ref. ^99^). In this picture, we are observing WD 1856 b in the very last, short-lived stage of high-eccentricity migration. Although we cannot rule it out based on the available data, we consider this explanation unlikely.

## Online content

Any methods, additional references, Nature Portfolio reporting summaries, source data, extended data, supplementary information, acknowledgements, peer review information; details of author contributions and competing interests; and statements of data and code availability are available at 10.1038/s41586-026-10514-7.

## Supplementary information

Peer Review file

## Source data

Source Data Fig. 1

Source Data Fig. 2

Source Data Extended Data Fig. 2

Source Data Extended Data Fig. 3

## Figures and Tables

**Extended Data Table 1:**
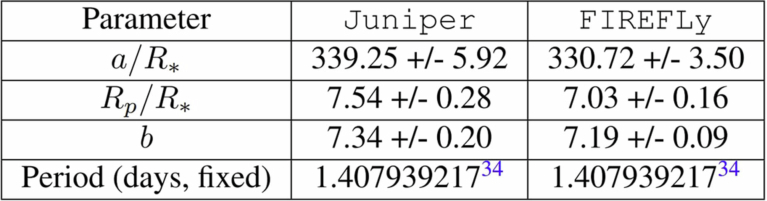
Best-fitting transit model parameters

The white light curve transit model parameters for the Juniper and FIREFLy data reductions are compared. We note that the planet–star radius ratio (*R*_p_/R_*_) and the impact parameter (*b*) are strongly correlated for a grazing transit. The period is fixed for both reductions and taken from ref. ^34^.

**Extended Data Table 2:**
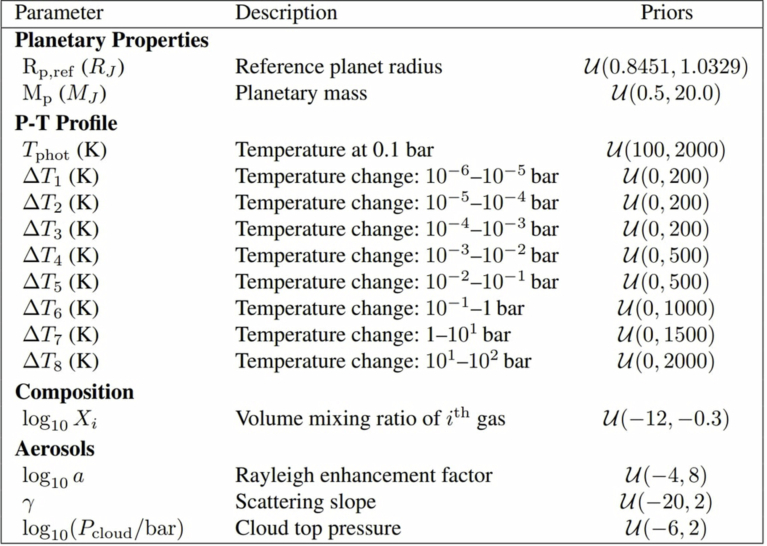
Atmospheric retrieval model parameters and priors

The free parameters defining the POSEIDON retrieval model used in the main text are listed. The volume mixing ratio parameters encode the chemical abundances of CH_4_, NH_3_, H_2_O, CO_2_, CO, HCN, C_2_H_2_, C_2_H_4_, C_2_H_6_, H_2_S and PH_3_. The prior for each parameter is uniformly distributed between a lower and upper limit, ${\mathcal{U}}({x}_{1},{x}_{2})$.

**Extended Data Table 3:**
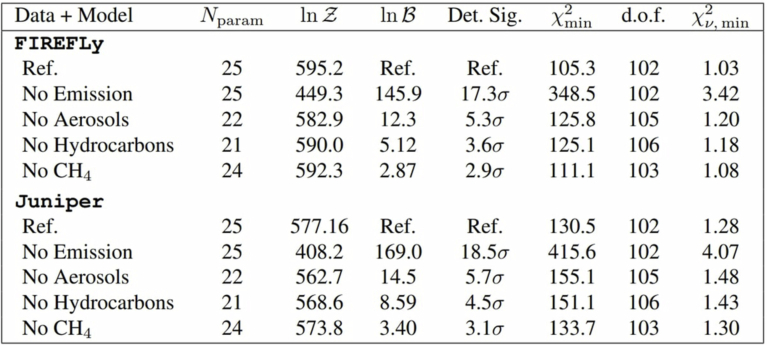
Atmospheric retrieval model statistics

Model statistics are listed for the 25-parameter reference POSEIDON model (the rows marked ‘Ref.’), alongside nested models without nightside thermal emission, without aerosols, without hydrocarbons and without CH_4_ for both the FIREFLy and Juniper data reductions. The model statistics are the number of free parameters (*N*_param_), the log-Bayesian evidence ($\mathrm{ln}{\mathcal{Z}}$), the log-Bayes factor ($\mathrm{ln}{\mathcal{B}}$), the equivalent detection significance (Det. Sig.), the chi-squared of the best-fit spectrum (${\chi }_{min}^{2}$), the degrees of freedom (d.o.f.) and the reduced chi-squared of the best-fit spectrum (${\chi }_{\nu ,min}^{2}$).

## Data Availability

The raw data from this study are available from the Space Science Telescope Institute’s Mikulski Archive for Space Telescopes (https://archive.stsci.edu/) under programme JWST-GO-2358. The FIREFLy and Juniper transmission spectra of WD 1856 b are available from Zenodo at 10.5281/zenodo.18200586 (ref. ^102^). Source data are provided with this paper.

## Code availability

POSEIDON is available at https://github.com/MartianColonist/POSEIDON. A Python script to reproduce the retrieval results is available from Zenodo at 10.5281/zenodo.18200586 (ref. ^102^). batman is available at https://github.com/lkreidberg/batman. emcee is available at https://github.com/dfm/emcee. ExoTiC-LD is available at https://github.com/Exo-TiC/ExoTiC-LD. MESA is available at https://zenodo.org/records/7983526 (ref. ^103^). FIREFLy and Juniper are not publicly available at present. Requests for further details on FIREFLy and Juniper should be addressed to D.K.S. (dsing@jhu.edu) and V.A.B. (vab55@cornell.edu), respectively.
