# Supplementary material for: Aerosols and hydrocarbons in the atmosphere of a white dwarf planet
Source: Nature. 2026 Jul 1;655(8121):76–80. doi: 10.1038/s41586-026-10514-7 (PMC13322981; doi:10.1038/s41586-026-10514-7)
Supplement: Supplementary file 1 — Peer Review file [file 41586_2026_10514_MOESM1_ESM.pdf]

nature portfolio

Peer Review File

Aerosols and hydrocarbons in the atmosphere of a white dwarf planet

Corresponding Author: Dr Ryan MacDonald

This file contains all reviewer reports in order by version, followed by all author rebuttals in order by version.

Version 0:

Reviewer comments:

Referee #1

(Remarks to the Author)
The authors present the detection of an atmosphere on the white dwarf planet WD 1856 b using transmission spectroscopy with JWST NIRSpec PRISM. This is the first time that transit spectroscopy has been used to characterise the atmosphere of a white dwarf planet. They find evidence for nightside thermal emission as well as methane and aerosols in the planet's atmosphere. They also use their observations to find the mass and effective temperature of their target. They incorporate this information into models to infer that the planet is likely to have undergone a reheating event, which has interesting implications for the evolution of the system.

This is a novel and exciting result, with important consequences for both exoplanet and white dwarf science. However, I would like to see a number of tests performed to ensure the robustness of the results presented in the paper before recommending it for publication. Detailed comments are included below.

ABSTRACT

In L053/054 the authors state that WD 1856 b's atmosphere has a similar CH4 abundance (~10%) to Neptune's deep atmosphere, citing Karkoschka & Tomasko 2011. My understanding is that this paper quotes a CH4 mixing ratio at deep layers of 4%±1% (left column, page 795). Is my reading of this paper correct? If not, please explain how you derived the ~10% value for Neptune. If the value for Neptune is indeed 4%, then I do not think that the values are close enough for this statement to be included in the abstract. This is compounded by the fact that if uncertainty across data reductions is included (see later comment), then the total uncertainty on the CH4 abundance for WD 1856 b rises to ±1 dex. A comparison with the atmosphere of Neptune could still be included in the main text at its present location.

MAIN TEXT

I am hesitant to agree that there is "broad consistency between retrieval results across the reductions" as stated in L131. To start, only the posteriors for 6 out of a total of 22 free parameters are shown anywhere in the manuscript, making it difficult to assess overall agreement. The authors should add a supplementary figure showing full posterior distributions for each retrieval. Furthermore, several marginalized posteriors derived from the Eureka! reduction do not agree with the other two reductions. For example, the retrieved CH4 abundance from Eureka! is 0.1-2%, compared with 4-23% from FIREFLy. The choice to focus on FIREFLy appears somewhat arbitrary, motivated only by the broader constraint on the mass obtained by this reduction. One could just as easily imagine choosing a different reduction due to broader constraints on a different parameter. Unless the agreement between reductions is improved, or a clearer argument is made to prefer one reduction over the others, the authors should place the different reductions on an equal footing, following the approach of e.g., Powell et al. (2024, Nature). Retrieved parameter values and associated uncertainties should then reflect the additional uncertainty between reductions. Detection significances should also be quoted for all reductions.

It is becoming standard practice to show multiple retrieval fits to JWST spectra to check for consistency (e.g. Taylor et al. 2023, MNRAS, Coulombe et al. 2023, Nature). The authors should apply at least one additional retrieval framework on their "fiducial" spectra and model setups (i.e. whatever goes in the main text). This is an important robustness test, particularly given the variable CH4 abundance between data reductions as well as between parametric cloud vs. Mie scattering models. I realise that the forward model has been adapted somewhat for this specific case to include nightside emission. However, a

number of codes exist that retrieve both transmission and emission spectra, allowing the same modification to be made.

The authors should comment on the overall quality of the fit to the data in the main text (paragraph starting at L134), and point out any regions of the spectrum that do not appear to be well-fit by their model. This should include an overall goodness-of-fit statistic for their best-fit model, such as reduced chi-squared, as well as a residuals plot showing the deviation between the best-fit model and the data, in units of sigma (this can be a supplementary figure). I am concerned about the model fit in some places. For example, in L168 the authors note that CH4 causes absorption bands near 1.7, 2.3 and 3.4 micron. However, the large feature near 3.4 micron does not appear to be well-fit by the model, with the peak in the data appearing at longer wavelengths than the CH4 peak from the models.

Figure 3a should be clarified to show how nightside emission contributes to the spectrum. At the moment it appears that the aerosols (green line) are the cause of the decrease in transit depth at long wavelengths, contradicting Figure 2a which attributes this to nightside emission.

The connection between the retrieved properties of the planet and its thermal history (L235 onwards) is intriguing. In a similar manner to the above comments, please discuss how this analysis changes based on the chosen data reduction - it is noted in L772 that this analysis only considers the FIREFLy reduction.

METHODS

The description of the FIREFLy reduction is considerably shorter than the other two reductions, and is written in a very different format, making it difficult to assess the differences between approaches. Like the other two descriptions, the description of FIREFLy should be modified to specify the different stages (1-5) in the reduction process. Are the “uncalibrated images” described in the FIREFLy reduction identical to the uncal.fits files from MAST? If so, they should be identified as such. They should also mention which limb darkening package they use (both other reductions use ExoTiC-LD, does FIREFLy use this as well?)

The measured average planet-star radius ratio, period, a/R*, and impact parameters should be reported for all reductions, as well as residuals. Some of these appear to be missing (with some quoted for FIREFLy in the main text and some quoted for the others in Methods).

Both the FIREFLy and Juniper reductions only use the wavelength range 0.5-3 microns to fit the broadband light curve, stating that the planetary emission at longer wavelengths could bias the transit depth. However, the Eureka! reduction appears to use the full wavelength range for this. The authors should either justify why they don’t expect this to bias their transit depths in the case of Eureka! or adjust the Eureka! reduction to avoid any possible bias.

The authors should discuss whether they considered patchy cloud models as well as the uniform cloud model presented in the paper (L738), and how these compared to the uniform cloud assumption. If patchy clouds were not considered, this decision should be justified.

Similarly, the authors should justify the choice of chemical species included in the retrieval. The list provided in L728 is not an exhaustive list of species that are typically included in retrievals: for example, HCN is not included. What were the motivating reasons behind including certain species and not others?

Minor comments/suggested edits:

In the caption for Figure 1, please indicate the average size of the error bars for the blue data points, since they are too small to be seen on the figure.

Please define the error bars shown in the transmission spectrum the first time it is shown (i.e. Fig 2). Are these just the ±1 sigma values of the fits to the transit depths, or is a uniform positive and negative error bar used?

STScI typically refers to the GO as “General Observing” rather than “Guest Observer”. I would suggest changing L080/L081 to reflect this.

It is interesting that such an unusual planet should be the first for which nightside thermal emission was detected. The manuscript could benefit from a brief discussion of whether the detection of nightside emission from this planet was expected, given its properties, and/or from referencing literature on the detectability of nightside emission from transiting exoplanets. If this literature does not exist, it would also be good to know this.

The authors should also comment briefly on the potential implications of the possible phosphine detection mentioned in L171 - is this a molecule that we might have expected to find in the atmosphere of this planet given past chemical modelling?

It is somewhat concerning that the thermal evolution models so significantly overpredict the planet’s radius, as shown in Extended Data Figure 9. While this is discussed in a good level of detail in the methods from L1099, a sentence mentioning this caveat should be added to the main text. It could also serve as an important motivator for future modelling work.

The authors should make the reduced spectra publicly available upon publication, alongside the raw data.

Referee #2

(Remarks to the Author)
The paper "The Atmosphere of a White Dwarf Planet" by MacDonald et al. presents JWST observations of the transiting planet WD 1856 b, and detects the thermal emission from the planetary nightside for the first time. The authors also find evidence of CH_4 and aerosols in the atmosphere. This is the first white dwarf planet to be characterized in detail, and the paper clearly deserves publication in Nature. The authors also find evidence of a reheating event and suggest that migration can explain the observed properties of this system. The paper is well written and clear. I have a few major comments and several minor comments. I hope these suggestions strengthen the conclusions of this paper.

Major comments:

- One of the main results of this paper is the detection of CH4 in the transmission spectrum. The authors compare planetary atmosphere models against to data to find a >5sigma detection of CH4. However, without the model comparison, I have a hard time seeing these features in Extended Data Figures 2 and 3. I suggest that the authors compare the chi2 from their best-fitting model fit to linear fit between 0.5 and ~3.4 micron. My main point is that how confident are you (without using a model fit) that the data are inconsistent with a flat line? I am not questioning the results shown in Figure 3, but I just wonder if one could make a stronger case that the data are inconsistent with "no features" in this wavelength range.

I have a harder time seeing the PH3 feature in the data. Given the errors in the transmission spectrum, I would argue that there is no evidence of a PH3 feature. The sub-panel for logPH3 in Figure 3 is cut at -10. Similarly, Extended Data Figure 5 also has a lower limit of -10 for logPH3. In the Eureka! fits, there seems to be a relatively high chance of logPH3 being at the -10 level, or even lower. These figures should be extended to even lower PH3 levels, and I would be very cautious of claiming even a tentative detection of PH3 in this case.

- Line 634: "Teff = 4920 K, log g = 8.05, and NH/NHe = 4.1. This solution yields an Hα line that extends 2% below the continuum, which is consistent with previously obtained optical spectroscopy [10] (not considered here in our fit)."

The best-fitting parameters from this work is very similar to the results from Alonso et al. (2021). However, in that work Alonso et al. assumed a pure H atmospheric composition, whereas here the authors demonstrate that the overall SED requires a He-rich atmosphere. Given the problems with fitting the SEDs of cool WDs (see for example section 2.1 in https://ui.adsabs.harvard.edu/abs/2024MNRAS.527.8687O/abstract) which the authors are clearly aware of, it would be interesting to show a comparison between the observed Halpha feature and the predictions from this He-rich model. He-rich DAs have broader lines, though at low temperatures NLTE effects are also important (for example, https://ui.adsabs.harvard.edu/abs/2021MNRAS.502.4972K/abstract). The authors should discuss any issues with matching the observed Halpha line with their He-rich models.

- Line 777: "We note that Teff is remarkably well-determined, with a 1 σ uncertainty of 5K (Teff = 408+5 −5 K), since the multiple CH4 bands detected in our NIRSpec PRISM data determines the relative amplitude of other CH4 features at longer wavelengths.

I have a hard time believing that one can determine the temperature of the planetary companion to 5 K precision. I understand that these are internal erors, but there could be systematic uncertainties that were not included in this calculation. I suggest a bootstrapping analysis of the transmission spectrum to verify that the internal errors are really this small.

Minor comments:

- Line 57: "Based on cooling models, these results require the planet to have undergone a significant reheating event 4.5+0.9 −1.1 Gyr (> 0.27 Gyr to 3 σ) after the red giant phase."

One cannot definitively say when the reheating event happened. These results do not require, but suggest the planet might have undergone a reheating event at that time. So I suggest revising this sentence.

- Line 70: "The host white dwarf is located at 25 pc and has a DC spectral class with an effective temperature of 4710 K, a mass of 0.0131 MSun, a radius of 0.0131 RSun,"

There are two problems here. One, the mass of the WD is given as 0.0131 Msun, which is obviously a typo. The second is that even though the discovery paper classified this object as a DC white dwarf, further observations by Alonso et al. (2021) detected an Halpha feature using GTC (also discussed above). Hence, this white dwarf is clearly a DA spectral class object.
See Figure 2 in
https://www.aanda.org/articles/aa/full_html/2021/05/aa40359-21/aa40359-21.html
Also see Figure 2 in
https://iopscience.iop.org/article/10.3847/1538-3881/ac2d26/pdf

- Line 83: "As the host white dwarf WD 1856 is a faint target" This is a relative statement, so I suggest "As the host white dwarf WD 1856 is a relatively faint target"

- Line 96: Rp/Rsun, b, and a/Rsun are presented with 3 digits after zero. Given the errors, it would be better to present these as, for example, a/Rsun = 330.7 +- 3.6 etc.

- Figure 2 left panel: I suggest flipping the y-axis in the left panel. Because this is the transit depth, when I see "Nightside Emission", I expect an emission feature, whereas the way the figure is displayed right now, the transmission spectrum dips. I know this is a minor detail, but I think it would help the reader visualize this better.

- Line 243: "10 Gyr age of the system" should be "~10 Gyr age of the system".
Also, the authors did not discuss how they calculated the total age of 10 Gyr for this system. There should be a mention of what initial-final mass relation is used, the progenitor mass +- error bars, and the total age with error bars.

- Line 250: "common-envelope evolution coincides with the end of the progenitor's red giant phase (5.4±0.7 Gyr ago; see Methods)"
- Line 259: "the cooling age of the host white dwarf (5.4±0.7) Gyr"

The WD cooling age represents the time it takes for a hot WD (Teff~100,000 K) to cool down to its current temperature. This is not necessarily the same as the end of the red giant phase. Obviously, the AGB phase does not take long, but the authors should at least mention how long it takes for WD 1856's progenitor to evolve from the end of the red giant phase to the beginning of the WD stage based on their MESA model.

- Line 314: "Furthermore, our detection of planetary thermal emission raises the prospect of directly detecting thermal emission from non-transiting planets orbiting white dwarfs with future JWST observations"

This is already done by Mullally et al. (2024). So I'm not sure why this statement is included.

- Line 355: "Several pre-transit exposures showed abnormally low flux levels, which we flagged as outliers and removed from the remaining analysis."

It would be beneficial to discuss the nature of these outliers. Was there a structure to these exposures? Where they back to back, or just observed randomly? Was the entire spectrum low, or is it in certain wavelenght regions? What is the evidence that these are not intrinsic to the source?

- Line 729: "The remainder of the atmosphere is composed of H2 and He with an abundance ratio of He/H2 = 0.17."

It would be beneficial to explain why this He/H2 ratio is chosen.

Version 1:

Reviewer comments:

Referee #1

(Remarks to the Author)
I appreciate the authors' efforts to address my comments. Overall, the latest version of the manuscript is very good. However, I have two concerns arising from the revisions that I would like to see addressed before recommending the paper for publication:

1. There has recently been substantial debate within the community regarding the use of "sigmas" to report the statistical significance of a detection of a chemical species or other atmospheric component. In particular, the sigma significance obtained following Benneke & Seager 2013, while frequently used in the exoplanet atmospheres community, has been shown to provide an overly optimistic assessment of the detection significance. Recent papers have argued for recalibrating the N sigma value to e.g. (N-1) sigma (Schmidt et al 2025), or to abandon it entirely and use Bayes factors due to its potential to mislead (Kipping and Benneke 2025). The authors clearly state which method they use to calculate "detection significances" (they follow the relation described in Benneke and Seager 2013), and also show the Bayes factors between different models in Extended Data Table 3, which is very helpful. It is fine to quote N sigma values following this method, as long as it is described clearly. However, the sigma values for hydrocarbons, CH4, aerosols and thermal emission are provided in the first paragraph of the main text without appropriate context. Given the potential for these values to mislead without clear acknowledgment of their caveats, I suggest that they are omitted from the summary paragraph. If the authors wish to describe their confidence in the identification of certain components, they could perhaps convert Bayes factors to weak/moderate/strong evidence using the Jeffreys' scale. However, it is important to be aware that any such conversion to sigmas or another metric is somewhat arbitrary, and the nuances are often lost on most readers, so I urge caution in any such conversion.

2. Page 2, L94, right column: I am pleased that the authors now include the reduced chi squared values for the best-fit model for each data reduction. The authors state that they achieve "an excellent fit to the data" for both reductions. However, the reduced chi squared is quite different for each reduction (1.03 for FIREFLy, 1.28 for Juniper). For 102 degrees of freedom, the standard deviation of the reduced chi squared distribution is approximately 0.14 (following Andrae et al 2010). This suggests that the best fit to the FIREFLy reduction is well within one standard deviation, whereas the best fit to the Juniper

reduction is discrepant with the data by around two standard deviations. This does not significantly impact the results of the paper, since there is good agreement between the posterior distributions of the retrievals on both reductions, but the statement at L94 should be replaced with a brief discussion on the different quality of fit for each reduction.

Referee #2

(Remarks to the Author)
I read the revised version of the manuscript and the authors' response. The authors have responded to all of my comments, and I have no further suggestions.

Note that the caption for Extended Data Fig 2 still refers to "Eureka! (pink squares)" even though those reductions were taken out of the paper.

Open Access This Peer Review File is licensed under a Creative Commons Attribution 4.0 International License, which permits use, sharing, adaptation, distribution and reproduction in any medium or format, as long as you give appropriate credit to the original author(s) and the source, provide a link to the Creative Commons license, and indicate if changes were made.
In cases where reviewers are anonymous, credit should be given to 'Anonymous Referee' and the source.
The images or other third party material in this Peer Review File are included in the article’s Creative Commons license, unless indicated otherwise in a credit line to the material. If material is not included in the article’s Creative Commons license and your intended use is not permitted by statutory regulation or exceeds the permitted use, you will need to obtain permission directly from the copyright holder.
To view a copy of this license, visit https://creativecommons.org/licenses/by/4.0/

Response to the Referees

We thank the referees for their patience during the process of revising our manuscript. We have now conducted all the tests recommended by the referees, as detailed below, which were valuable suggestions to establish the fidelity of our results.

We note here the most significant changes to our manuscript:

1). We have re-run our retrieval analysis with a new opacity database to address the potential presence of other hydrocarbons near 3.4 um. Our main results are unchanged, but we do find that including C_2H_6 (as suggested by referee #1) does improve our model fits.

2). While we find good consistency between the FIREFLy and Juniper data reductions, the retrieval for our Eureka! reduction resulted in an unphysical solution when including C_2H_6 (2.6% of the atmosphere being C_2H_6, when this trace molecule cannot be more abundant that CH_4). We have since revisited the Eureka! reduction and identified several potential issues (the code has improved significantly in the last year), so we ultimately decided to drop the Eureka! reduction for the purposes of this study.

3). We have adopted the suggestion from referee #1 to present equally our retrieval results for both the data reductions (FIREFLy and Juniper) throughout the manuscript, rather than choosing a default reduction. The abstract and Main text now quote the 1 sigma range spanning both data reductions for atmospheric properties and the planetary mass.

We proceed to respond to each of the referee comments in turn.

Referee #1:

The authors present the detection of an atmosphere on the white dwarf planet WD 1856 b using transmission spectroscopy with JWST NIRSpec PRISM. This is the first time that transit spectroscopy has been used to characterise the atmosphere of a white dwarf planet. They find evidence for nightside thermal emission as well as methane and aerosols in the planet's atmosphere. They also use their observations to find the mass and effective temperature of their target. They incorporate this information into models to infer that the planet is likely to have undergone a reheating event, which has interesting implications for the evolution of the system.

This is a novel and exciting result, with important consequences for both exoplanet and white dwarf science. However, I would like to see a number of tests performed to ensure the robustness of the results presented in the paper before recommending it for publication. Detailed comments are included below.

We thank the referee for their suggested robustness tests, which we have completed as described below.

ABSTRACT

In L053/054 the authors state that WD 1856 b's atmosphere has a similar CH4 abundance (~10%) to Neptune's deep atmosphere, citing Karkoschka & Tomasko 2011. My understanding is that this paper quotes a CH4 mixing ratio at deep layers of 4%±1% (left column, page 795). Is my reading of this paper correct? If not, please explain how you derived the ~10% value for Neptune. If the value for Neptune is indeed 4%, then I do not think that the values are close enough for this statement to be included in the abstract. This is compounded by the fact that if uncertainty across data reductions is included (see later comment), then the total uncertainty on the CH4 abundance for WD 1856 b rises to ±1 dex. A comparison with the atmosphere of Neptune could still be included in the main text at its present location.

While Neptune's CH_4 abundance (4% -> log CH_4 = -1.40) is consistent with the values we retrieve for WD 1856b within 1 sigma (log CH_4 = -1.17(+0.45)(-0.52) for FIREFLy and -1.11(+0.41)(-0.45) for Juniper), we agree with the referee that the previously quoted value of ~ 10% for Neptune (derived as the nearest exponent for CH_4 in logarithmic units) could risk misstating the conclusions of Karkoschka & Tomasko (2011). Therefore, we have removed this statement from the abstract and have focused the comparison with Neptune's CH_4 abundance in the Main text.

MAIN TEXT

I am hesitant to agree that there is "broad consistency between retrieval results across the reductions" as stated in L131. To start, only the posteriors for 6 out of a total of 22 free parameters are shown anywhere in the manuscript, making it difficult to assess overall agreement. The authors should add a supplementary figure showing full posterior distributions for each retrieval.

We have added a new Extended Data Figure showing the full posterior distributions for both data reductions, with all 25 free parameters shown (the three additional parameters are the abundances of HCN, C_2H_4, and C_2H_6), also reproduced below. As can be seen, the agreement between FIREFLy and Juniper is excellent with all parameters consistent within 1 sigma.

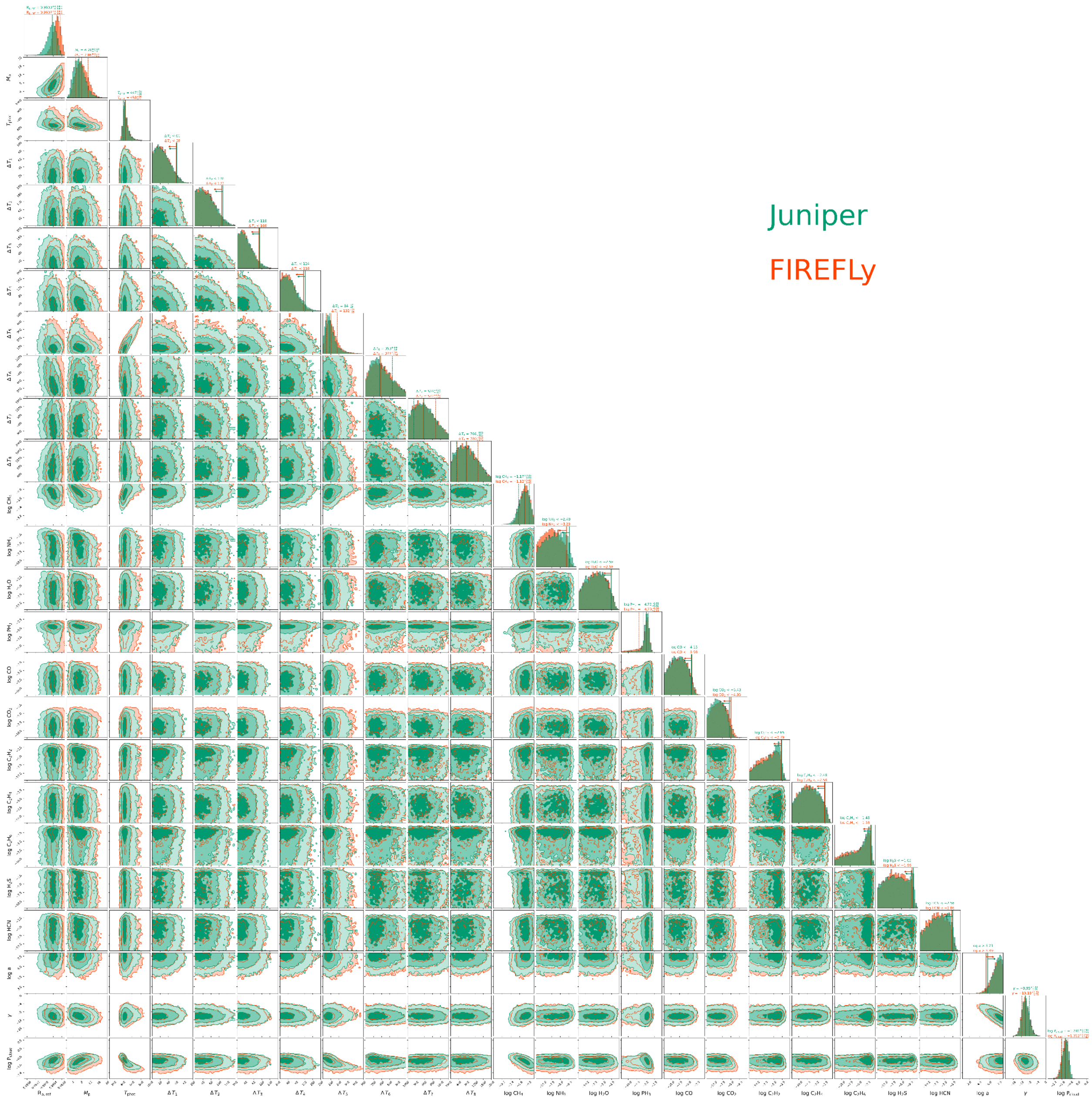

Furthermore, severbal marginalized posteriors derived from the Eureka! reduction do not agree with the other two reductions. For example, the retrieved CH4 abundance from Eureka! is 0.1-2%, compared with 4-23% from FIREFLy. The choice to focus on FIREFLy appears somewhat arbitrary, motivated only by the broader constraint on the mass obtained by this reduction. One could just as easily imagine choosing a different reduction due to broader constraints on a different parameter. Unless the agreement between reductions is improved, or a clearer argument is made to prefer one reduction over the others, the authors should place the different reductions on an equal footing, following the approach of e.g., Powell et al. (2024,

Nature). Retrieved parameter values and associated uncertainties should then reflect the additional uncertainty between reductions. Detection significances should also be quoted for all reductions.

We agree with the referee that the most conservative approach is to fold the uncertainty from the different data reductions into our quoted atmospheric properties. Per the referee's suggestion, we have adopted the approach from Powell et al. (2024) by quoting the range of atmospheric properties spanning the two data reductions.

With this in mind, we have made the following changes to our manuscript:

1). The quoted atmospheric properties for WD 1856b in our manuscript (e.g. in the abstract) now span the 1 sigma credible intervals of the retrieved parameters from the two data reductions.

2). Figure 3 now shows the posterior distributions from both data reductions (reproduced below). We also added the posterior of the retrieved aerosol scattering slope (gamma) to Figure 3. With this change, the previous Extended Data Figure 5 (showing the sensitivity to different reductions unnecessary) is redundant and has been removed.

3). The detection significances for both reductions are now quoted, with the variation in these significances quoted in the abstract as a range.

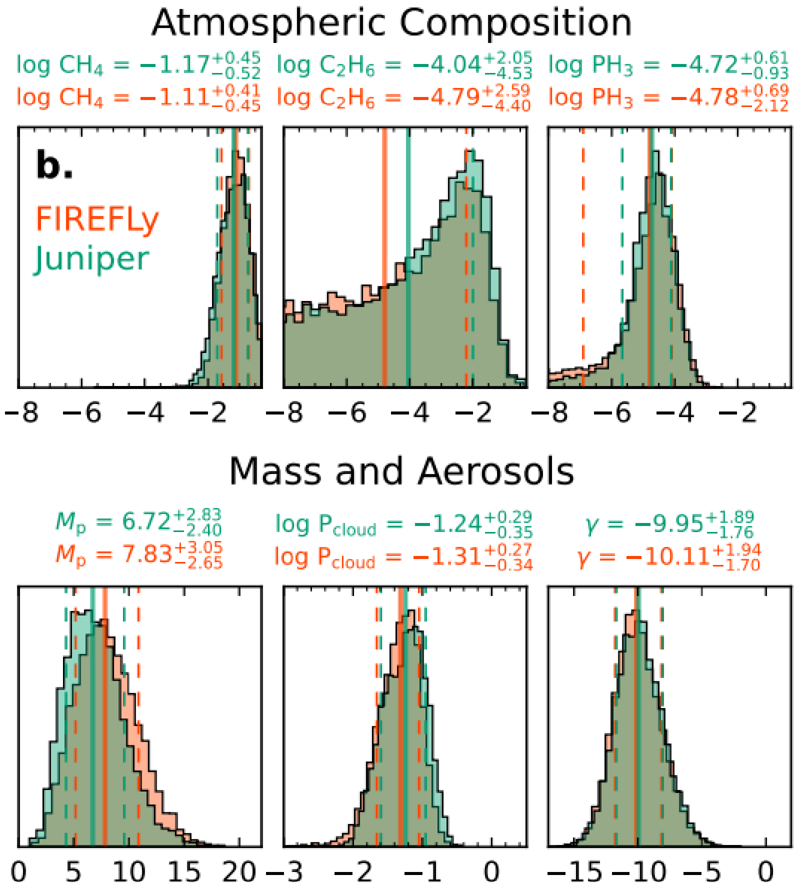

It is becoming standard practice to show multiple retrieval fits to JWST spectra to check for consistency (e.g. Taylor et al. 2023, MNRAS, Coulombe et al. 2023, Nature). The authors should apply at least one additional retrieval framework on their “fiducial” spectra and model setups (i.e. whatever goes in the main text). This is an important robustness test, particularly given the variable CH4 abundance between data reductions as well as between parametric cloud vs. Mie scattering models. I realise that the forward model has been adapted somewhat for this specific case to include nightside emission. However, a number of codes exist that retrieve both transmission and emission spectra, allowing the same modification to be made.

While we agree with the general point of the referee about retrieval code intercomparisons, the modifications we made to the transmission spectrum radiative transfer module in POSEIDON to handle the grazing geometry of WD 1856b are highly non-trivial. There are no other open source retrieval codes that have the functionality to handle retrievals for this grazing transit geometry. More specifically, the forward models in other open source retrieval codes for transmission spectra explicitly assume the complete overlap of the planet and its atmosphere with the host star, which is not the case for WD 1856b. Relaxing this assumption requires a fundamental code re-engineering of the standard integral approach used to calculate transmission spectra. It is beyond the scope of our manuscript to rewrite the underlying forward model of another retrieval code to handle grazing transit geometries.

Furthermore, we note that many published papers have included POSEIDON as part of a retrieval code comparison, with all codes finding excellent agreement (within 1 sigma). The two cited papers, Taylor+2023 and Coulombe+2023, both included POSEIDON retrieval results that were consistent with the other codes. Additional recent examples showing the agreement of POSEIDON’s results with other retrieval codes include Grant+2023, Fournier-Tondreau+2024, Gressier+2024, and Bello-Arufe+2025. The well-documented agreement of POSEIDON with other retrieval codes demonstrates that common retrieval code model differences (e.g. cross section differences) are unlikely to impact our atmospheric inferences for WD 1856b.

The authors should comment on the overall quality of the fit to the data in the main text (paragraph starting at L134), and point out any regions of the spectrum that do not appear to be well-fit by their model. This should include an overall goodness-of-fit statistic for their best-fit model, such as reduced chi-squared, as well as a residuals plot showing the deviation between the best-fit model and the data, in units of sigma (this can be a supplementary figure).

We thank the referee for raising these points. We now quote reduced chi^2 values in the Main text (which are close to 1 for both reductions; 1.03 for FIREFLy, and 1.28 for Juniper) and provide a new Extended Data Table 3 with all the model statistics. Per the referee’s suggestion, we have also added a residuals panel to Figure 3 for the best-fitting model. We also added residuals panels to Figure 1 (the transit light curves) and Extended data Figure 3 (the WD host spectrum).

I am concerned about the model fit in some places. For example, in L168 the authors note that CH4 causes absorption bands near 1.7, 2.3 and 3.4 micron. However, the large feature near 3.4 micron does not appear to be well-fit by the model, with the peak in the data appearing at longer wavelengths than the CH4 peak from the models.

We agree with the referee that the potential presence of a feature at 3.4 um is intriguing. From the solar system, there is a similar feature at 3.4 um seen in solar occultation spectra of Saturn (Dalba+2015, ApJ), which Dalba+2015 attributes to a C-H stretching mode of an unknown hydrocarbon. As discussed in a response below, the addition of C_2H_6 to our retrievals has improved the fit quality at 3.4 um (see Figure 3, also reproduced below) while leaving our previous inference of CH_4 relatively unchanged.

We have now performed additional robustness tests to confirm the presence of CH_4. First, we computed a new CH_4 cross section using the state-of-the-art ExoMol ‘MM’ line list (Yurchenko+2024), which supersedes the ‘34to10’ line list (Yurchenko+2017) used in our previous retrievals. Second, we added additional hydrocarbon species (HCN, C_2H_4, and C_2H_6) with absorption features near 3.4 um (as the referee suggested below) to avoid overestimating the CH_4 detection significance. Third, we conducted additional retrievals with the data from 3.1 to 3.5 um removed to confirm that the CH_4 inference is not driven by outlier points near 3.4 um.

The inclusion of additional hydrocarbons lowers the CH_4 detection significance for both FIREFLY and Juniper to 3σ. However, the combined significance of *any* hydrocarbon is ~ 4σ (which we now quote in the abstract). Our retrieval on the FIREFLy data with 3.1-3.5 um masked returns a CH_4 detection significance of 3.0σ. Therefore, we are confident that the inference of CH_4 (and hydrocarbons more generally) is not driven by outlier points near 3.4 um We added a stance to the Main text regarding the data cut test.

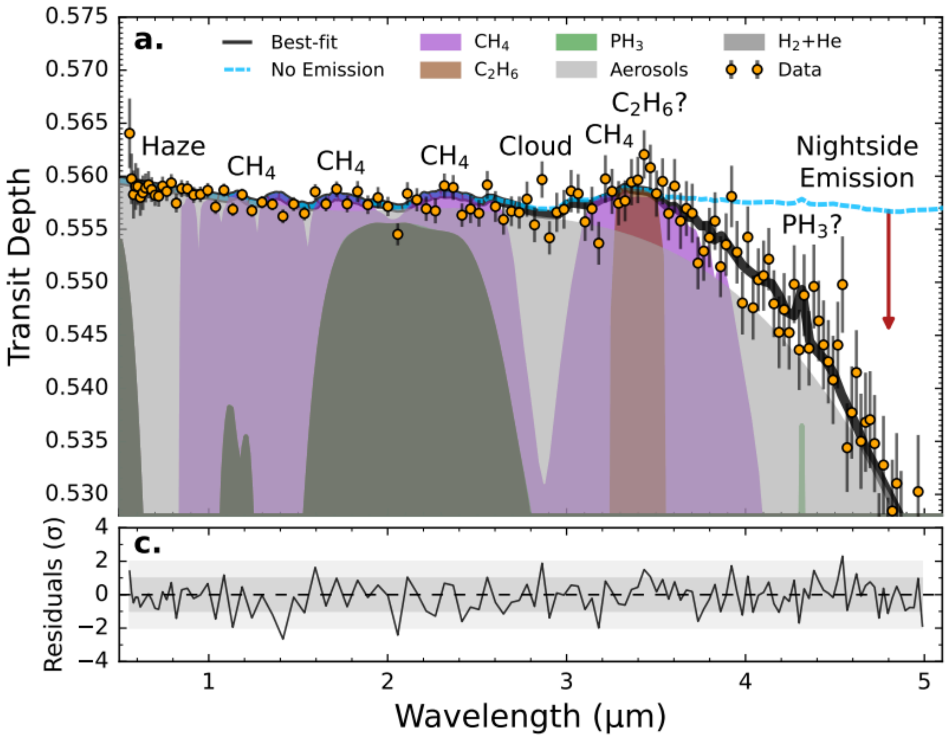

Figure 3a should be clarified to show how nightside emission contributes to the spectrum. At the moment it appears that the aerosols (green line) are the cause of the decrease in transit depth at long wavelengths, contradicting Figure 2a which attributes this to nightside emission.

We agree with the referee that visualising the impact of nightside emission on this transmission spectrum can be counter-intuitive. We have therefore added another model to Figure 3 showing the best-fitting model with nightside emission disabled (blue dashed line in the reproduction above). We also modified the spectral decomposition in Figure 3 to use solid shading for the contributions and added the H_2 continuum, which better highlights the contribution from the aerosol continuum between CH_4 bands. This demonstrates that the aerosols explain both the short wavelength scattering slope and the flat region from 2.6-3.1 um between the CH_4 bands.

The connection between the retrieved properties of the planet and its thermal history (L235 onwards) is intriguing. In a similar manner to the above comments, please discuss how this analysis changes based on the chosen data reduction - it is noted in L772 that this analysis only considers the FIREFLy reduction.

We now include both the FIREFLy and Juniper T_{eff} histograms in Extended Data Figure 5 and the t_0 posteriors and evolutionary histories for both reductions in Figure 4 and Extended data Figures 7 and 8. The abstract, Main text, and Methods now provide the ranges for these quantities spanning both data reductions.

METHODS

The description of the FIREFLy reduction is considerably shorter than the other two reductions, and is written in a very different format, making it difficult to assess the differences between approaches. Like the other two descriptions, the description of FIREFLy should be modified to specify the different stages (1-5) in the reduction process.

We have adopted the referee's suggestion by expanding the FIREFLy reduction description in the Methods to cover each stage of the reduction process.

Are the “uncalibrated images” described in the FIREFLy reduction identical to the uncal.fits files from MAST? If so, they should be identified as such. They should also mention which limb darkening package they use (both other reductions use ExoTiC-LD, does FIREFLy use this as well?)

Indeed, the uncalibrated images are referring to the uncal.fits files from MAST. The text has been updated to clarify this as well as the use of ExoTiC-LD for limb-darkening.

The measured average planet-star radius ratio, period, a/R*, and impact parameters should be reported for all reductions, as well as residuals. Some of these appear to be missing (with some quoted for FIREFLy in the main text and some quoted for the others in Methods).

Extended Data Table 1 now reports these quantities for the two data reductions.

Both the FIREFLy and Juniper reductions only use the wavelength range 0.5-3 microns to fit the broadband light curve, stating that the planetary emission at longer wavelengths could bias the transit depth. However, the Eureka! reduction appears to use the full wavelength range for this. The authors should either justify why they don't expect this to bias their transit depths in the case of Eureka! or adjust the Eureka! reduction to avoid any possible bias.

As mentioned above, the Eureka! Reduction is no longer included in the manuscript. Extended data Figure 2 shows the good agreement between the FIREFLy and Juniper reductions, while the other Figures in the manuscript demonstrate the excellent agreement in the retrieval results for these two reductions.

The authors should discuss whether they considered patchy cloud models as well as the uniform cloud model presented in the paper (L738), and how these compared to the uniform cloud assumption. If patchy clouds were not considered, this decision should be justified.

Due to WD 1856b's unique grazing transit geometry with Rp/Rs > 1, only a small fraction of the planetary terminator overlaps the star. Consequently, the transmission spectrum is localised to a small region of WD 1856b's atmosphere (in a similar manner to how solar or stellar occultations observed by spacecraft probe a specific line of sight through an atmosphere). Our use of a uniform cloud model is therefore not equivalent to assuming that the entire terminator has a uniform cloud deck. Rather, our model assumes that the small fraction of the terminator we probe has homogeneous cloud properties. We have added this justification to the Methods.

Similarly, the authors should justify the choice of chemical species included in the retrieval. The list provided in L728 is not an exhaustive list of species that are typically included in retrievals: for example, HCN is not included. What were the motivating reasons behind including certain species and not others?

Our retrievals include the main chemical species expected at WD 1856b's temperature. Since WD 1856b is substantially cooler than the planets usually probed by transmission spectroscopy (e.g. hot Jupiters), many chemical species that can matter for hotter planets will not exist in the gas phase at this temperature (e.g. Na, K, and metal oxides).

However, we have now re-run all our retrievals with the addition of HCN, C_2H_4, and C_2H_6, the latter two of which were recently added to POSEIDON's opacity database (Mullens+2024). The plot below (for the FIREFLy reduction) shows the inference of CH_4 remains with these three new hydrocarbons included (alongside the potential evidence for PH_3). HCN and C_2H_4 are not detected, but we do see a potential hint of C_2H_6, due to the absorption of this hydrocarbon near 3.4 um (e.g. Figure 3). The manuscript has been updated throughout using this new retrieval model including these three new hydrocarbons.

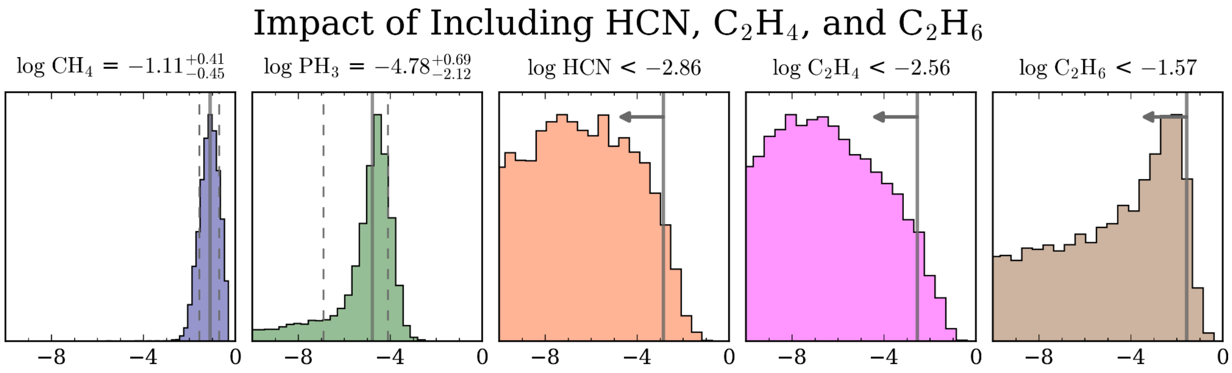

Minor comments/suggested edits:

In the caption for Figure 1, please indicate the average size of the error bars for the blue data points, since they are too small to be seen on the figure.

We now quote the average error bar sizes in the caption of Figure 1.

Please define the error bars shown in the transmission spectrum the first time it is shown (i.e. Fig 2). Are these just the ±1 sigma values of the fits to the transit depths, or is a uniform positive and negative error bar used?

We have updated the caption of Figure 2 to specify that the error bars are ±1 sigma values.

STScI typically refers to the GO as “General Observing” rather than “Guest Observer”. I would suggest changing L080/L081 to reflect this.

We have confirmed that STScI uses “General Observer” and updated the terminology in our manuscript.

It is interesting that such an unusual planet should be the first for which nightside thermal emission was detected. The manuscript could benefit from a brief discussion of whether the detection of nightside emission from this planet was expected, given its properties, and/or from referencing literature on the detectability of nightside emission from transiting exoplanets. If this literature does not exist, it would also be good to know this.

We agree with the referee that the high-significance detection of nightside emission for WD 1856b was rather surprising (especially given the low equilibrium temperature). A recent paper by Kappelmier+2024 investigated the detectability of nightside thermal emission across the exoplanet population and presented the ‘top 10’ planets with the highest predicted nightside emission. To our knowledge, this new study (published after our manuscript was submitted) is the only literature on the detectability of nightside thermal emission in low-resolution transmission spectra and we now cite this study. WD 1856b was not predicted by

Kappelmier+2024 to have detectable nightside emission, since that study used a temperature similar to the equilibrium temperature (while our transmission spectrum shows WD 1856b has a much higher atmospheric temperature). The reason this 'unusual' planet has unexpectedly prominent nightside thermal emission is the combination of its high planet-star area ratio and the planetary temperature significantly exceeding its equilibrium temperature. One can therefore directly tie the detection of any nightside emission to our inference of a past re-heating event.

The authors should also comment briefly on the potential implications of the possible phosphine detection mentioned in L171 - is this a molecule that we might have expected to find in the atmosphere of this planet given past chemical modelling?

Jupiter's upper atmosphere contains PH_3 (e.g. Larson+1977), which is sustained via vertical mixing at a much higher abundance than expected under equilibrium predictions. A similar disequilibrium mechanism could well be at play in WD 1856b's atmosphere, which motivated the inclusion of PH_3 our the retrievals, and is now mentioned in the Main text. Additional chemical modelling of WD 1856b in future studies will help to elucidate this. However, given the long tail in the PH_3 abundance posteriors, we caution that additional observations will be required to confirm if PH_3 is present in WD 1856b's atmosphere.

It is somewhat concerning that the thermal evolution models so significantly overpredict the planet's radius, as shown in Extended Data Figure 9. While this is discussed in a good level of detail in the methods from L1099, a sentence mentioning this caveat should be added to the main text. It could also serve as an important motivator for future modelling work.

We have now added the requested sentence to the main text. The updated Extended data Figure 8 shows that this radius discrepancy is only at 2σ for the FIREFLy reduction.

The authors should make the reduced spectra publicly available upon publication, alongside the raw data.

All reduced transmission spectra and the raw JWST data will be publicly available in a Zenodo repository upon publication of the manuscript.

<u>Referee #2:</u>

The paper "The Atmosphere of a White Dwarf Planet" by MacDonald et al. presents JWST observations of the transiting planet WD 1856 b, and detects the thermal emission from the planetary nightside for the first time. The authors also find evidence of CH4 and aerosols in the atmosphere. This is the first white dwarf planet to be characterized in detail, and the paper clearly deserves publication in Nature. The authors also find evidence of a reheating event and suggest that migration can explain the observed properties of this system. The paper is well

written and clear. I have a few major comments and several minor comments. I hope these suggestions strengthen the conclusions of this paper.

We thank the referee for their valuable suggestions and feedback, which has strengthened the robustness of our results.

Major comments:

- One of the main results of this paper is the detection of CH4 in the transmission spectrum. The authors compare planetary atmosphere models against to data to find a >5sigma detection of CH4. However, without the model comparison, I have a hard time seeing these features in Extended Data Figures 2 and 3. I suggest that the authors compare the chi2 from their best-fitting model fit to linear fit between 0.5 and ~3.4 micron. My main point is that how confident are you (without using a model fit) that the data are inconsistent with a flat line? I am not questioning the results shown in Figure 3, but I just wonder if one could make a stronger case that the data are inconsistent with "no features" in this wavelength range.

We have conducted the test outlined by the referee (see the plot below) for the FIREFLy data. We find that a flat line model has a chi^2 with respect to the data below 3.4 um of 83.75, compared to 63.49 for our retrieval best-fit. The corresponding reduced chi^2 are 1.196 for the flat line and 1.008 for the best-fitting atmosphere model (neglecting 14 redundant non-constrained parameters from the P-T profile and non-constrained molecular abundances).

The plot below demonstrates that the combination of aerosol opacity (the short wavelength slope) and hydrocarbon absorption (multiple CH_4 bands, alongside a potential C_2H_6 band at 3.4 micron present in this best-fitting model) is indeed a better fit than a flat line to the < 3.4 um data.

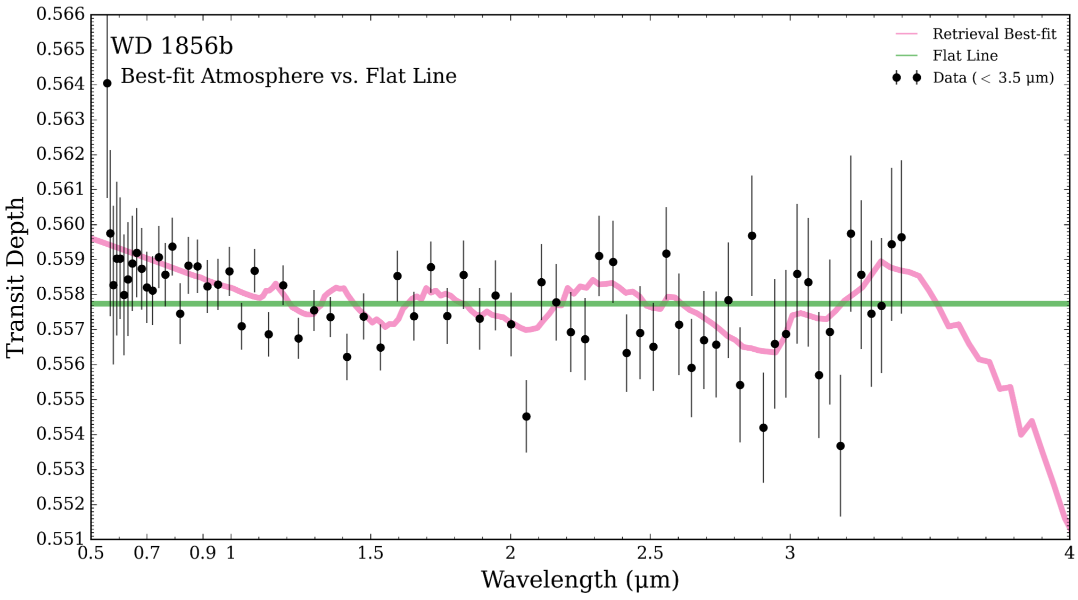

I have a harder time seeing the PH3 feature in the data. Given the errors in the transmission spectrum, I would argue that there is no evidence of a PH3 feature. The sub-panel for logPH3 in Figure 3 is cut at -10. Similarly, Extended Data Figure 5 also has a lower limit of -10 for logPH3. In the Eureka! fits, there seems to be a relatively high chance of logPH3 being at the -10 level, or even lower. These figures should be extended to even lower PH3 levels, and I would be very cautious of claiming even a tentative detection of PH3 in this case.

We agree with the referee that the evidence for PH_3 is marginal, and therefore we have carefully updated the terminology in our manuscript. Regarding the prior limits on the mixing ratios, we allow log mixing ratios down to -12 for all gases. The histograms in Figure 3 show mixing ratios down to -8 for clarity, but the full corner plots in the new Extended Data Figure 4 show the entire prior range. We have also added a new Extended Data Table 2 specifying all the prior ranges and a new Extended Data Table 3 providing the retrieval model statistics.

- Line 634: "Teff = 4920 K, log g = 8.05, and NH/NHe = 4.1. This solution yields an Hα line that extends 2% below the continuum, which is consistent with previously obtained optical spectroscopy [10] (not considered here in our fit)."

The best-fitting parameters from this work is very similar to the results from Alonso et al. (2021). However, in that work Alonso et al. assumed a pure H atmospheric composition, whereas here the authors demonstrate that the overall SED requires a He-rich atmosphere. Given the problems with fitting the SEDs of cool WDs (see for example section 2.1 in https://ui.adsabs.harvard.edu/abs/2024MNRAS.527.8687O/abstract) which the authors are clearly aware of, it would be interesting to show a comparison between the observed Halpha feature and the predictions from this He-rich model. He-rich DAs have broader lines, though at low temperatures NLTE effects are also important (for example, https://ui.adsabs.harvard.edu/abs/2021MNRAS.502.4972K/abstract). The authors should discuss any issues with matching the observed Halpha line with their He-rich models.

We thank the referee for highlighting this. Below we show a plot comparing the GTC spectrum of Alonso et al. (2021) to our best-fitting model to the JWST spectroscopy. The predicted H-alpha feature is not too broad compared to the observations. The model H-alpha feature is slightly too shallow compared to the GTC spectrum (~2% compared to ~3% deep), but we note that in the GMOS data from Xu et al. (2021) the H-alpha feature is also ~2% deep (see their Figure 2), as already stated in our manuscript. We therefore conclude that our mixed H+He solution is compatible with the existing optical spectroscopy.

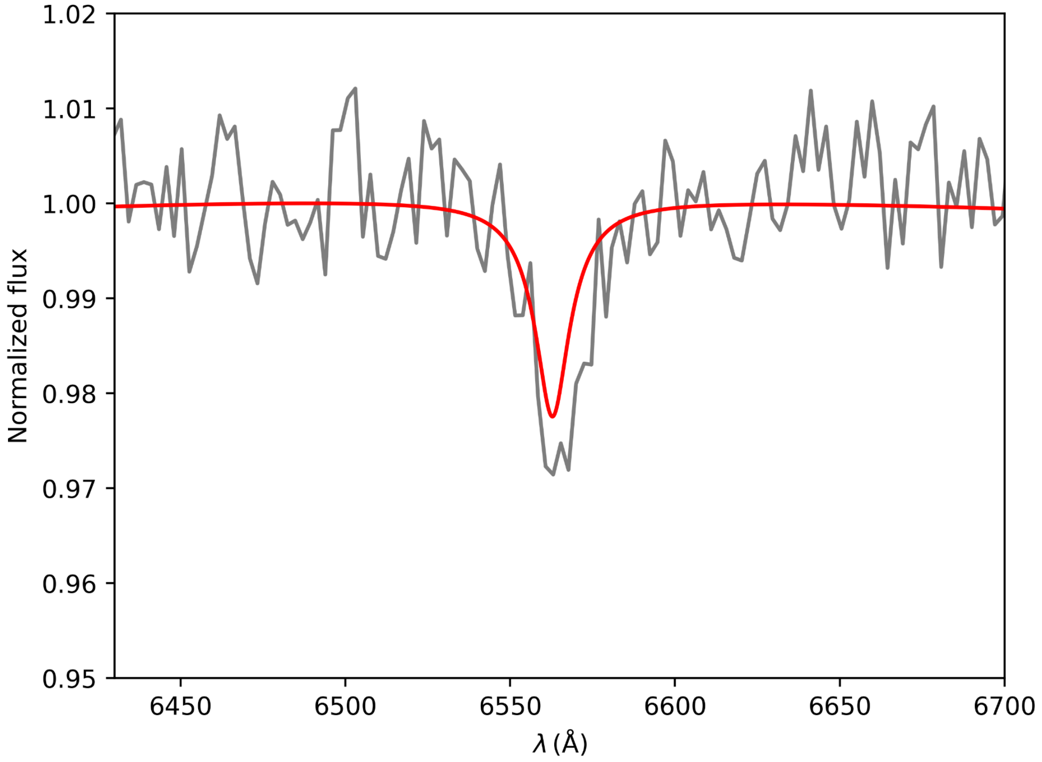

- Line 777: "We note that Teff is remarkably well-determined, with a 1 σ uncertainty of 5K (Teff = 408+5 −5 K), since the multiple CH4 bands detected in our NIRSpec PRISM data determines the relative amplitude of other CH4 features at longer wavelengths.

I have a hard time believing that one can determine the temperature of the planetary companion to 5 K precision. I understand that these are internal erors, but there could be systematic uncertainties that were not included in this calculation. I suggest a bootstrapping analysis of the transmission spectrum to verify that the internal errors are really this small.

Our updated retrieval analysis, now accounting for the uncertainty from different data reductions, results in a 1 sigma range for T_{eff} spanning 22 K (390-412 K). We note that this temperature is compatible with the previous retrieval results, even after adding a new molecular opacity that slightly improves the model fit (C_2H_6). This demonstrates that the overall impact on the retrieved effective temperature from both data-level choices and model-level choices is small, and we therefore see the quoted 22 K range in our revised manuscript as sufficiently conservative.

Minor comments:

- Line 57: "Based on cooling models, these results require the planet to have undergone a significant reheating event 4.5+0.9 −1.1 Gyr (> 0.27 Gyr to 3 σ) after the red giant phase."

One cannot definitively say when the reheating event happened. These results do not require, but suggest the planet might have undergone a reheating event at that time. So I suggest revising this sentence.

We have revised this sentence following the referee's suggestion.

- Line 70: "The host white dwarf is located at 25 pc and has a DC spectral class with an effective temperature of 4710 K, a mass of 0.0131 MSun, a radius of 0.0131 RSun,"

There are two problems here. One, the mass of the WD is given as 0.0131 Msun, which is obviously a typo. The second is that even though the discovery paper classified this object as a DC white dwarf, further observations by Alonso et al. (2021) detected an Halpha feature using GTC (also discussed above). Hence, this white dwarf is clearly a DA spectral class object. See Figure 2 in
https://www.aanda.org/articles/aa/full_html/2021/05/aa40359-21/aa40359-21.html
Also see Figure 2 in
https://iopscience.iop.org/article/10.3847/1538-3881/ac2d26/pdf

We thank the referee for catching these errors, which have now been fixed.

- Line 83: "As the host white dwarf WD 1856 is a faint target" This is a relative statement, so I suggest "As the host white dwarf WD 1856 is a relatively faint target"

Updated.

- Line 96: Rp/Rsun, b, and a/Rsun are presented with 3 digits after zero. Given the errors, it would be better to present these as, for example, a/Rsun = 330.7 +- 3.6 etc.

We have updated the quoted precision.

- Figure 2 left panel: I suggest flipping the y-axis in the left panel. Because this is the transit depth, when I see "Nightside Emission", I expect an emission feature, whereas the way the figure is displayed right now, the transmission spectrum dips. I know this is a minor detail, but I think it would help the reader visualize this better.

The standard field convention in transmission spectra papers is to have increasing transit depth on the y axis (so absorption is 'up' and emission is 'down'). We recognise this is the opposite convention from spectra in many other fields of astronomy, but since almost all exoplanet transmission spectra papers from the last 15 years follow this convention we have kept the

usual y axis convention. However, we have made the impact of nightside emission clearer via updates to Figure 3.

- Line 243: "10 Gyr age of the system" should be "~10 Gyr age of the system".
Also, the authors did ot discuss how they calculated the total age of 10 Gyr for this system. There should be a mention of what initial-final mass relation is used, the progenitor mass +- error bars, and the total age with error bars.

Our originally quoted 10 Gyr system age was from Vanderburg et al. (2020), who derived this estimate as an upper limit from thin disk kinematics. We have now computed an updated system age, 9.4(+2.5)(-1.9) Gyr, as outlined in the Methods section 'Host Progenitor and White Dwarf'. We provide the progenitor mass and uncertainties in this new paragraph.

- Line 250: "common-envelope evolution coincides with the end of the progenitor's red giant phase (5.4±0.7 Gyr ago; see Methods)"
- Line 259: "the cooling age of the host white dwarf (5.4±0.7) Gyr"

The WD cooling age represents the time it takes for a hot WD (Teff~100,000 K) to cool down to its current temperature. This is not necessarily the same as the end of the red giant phase. Obviously, the AGB phase does not take long, but the authors should at least mention how long it takes for WD 1856's progenitor to evolve from the end of the red giant phase to the beginning of the WD stage based on their MESA model.

We thank the referee for raising this important point of clarification. We have used MIST models to calculate the duration of the AGB phase, finding that the thermally pulsing AGB phase lasts 1.55 (+0.26)(-0.10) Myr and the pre-WD stage before cooling to 100,000 K lasts 33.8 (+52.7) (-2.5) kyr. These durations are now mentioned in the Methods, and we have carefully updated our manuscript to be clear when we are referring to the end of the AGB phase.

- Line 314: "Furthermore, our detection of planetary thermal emission raises the prospect of directly detecting thermal emission from non-transiting planets orbiting white dwarfs with future JWST observations"

This is already done by Mullally et al. (2024). So I'm not sure why this statement is included.

We have removed this statement.

- Line 355: "Several pre-transit exposures showed abnormally low flux levels, which we flagged as outliers and removed from the remaining analysis."

It would be beneficial to discuss the nature of these outliers. Was there a structure to these exposures? Where they back to back, or just observed randomly? Was the entire spectrum low, or is it in certain wavelenght regions? What is the evidence that these are not intrinsic to the source?

Upon inspection, the outliers are associated with clusters of bright/hot pixels which uniquely appear in an integration. These are very likely snowball events (https://jwst-docs.stsci.edu/known-issues-with-jwst-data/shower-and-snowball-artifacts#gsc.tab=0) which affect the up-the-ramp fit. As they appear in clusters, they often escape cosmic ray cleaning algorithms. Given only two exposures are affected, we elected to remove them from the TSO analysis.

- Line 729: "The remainder of the atmosphere is composed of H2 and He with an abundance ratio of He/H2 = 0.17."

It would be beneficial to explain why this He/H2 ratio is chosen.

Our adopted H_2/He ratio of 0.17 is broadly representative of the average ratio for the giant planets in the solar system (e.g. Atreya et al. 2020). We now state this.

Editor:

Dear Dr MacDonald

First of all, let me apologize for the unusually long time to get a decision to you. New submissions have been very high for the past four months, during which time I was buying a house in Nova Scotia and preparing my house in Maryland for sale (I will be retiring in late July and moving to Halifax).

Your manuscript, "The Atmosphere of a White Dwarf Planet", has now been seen by 2 referees, whose comments are attached below. While they find your work of interest, they have raised points that need to be addressed before we can make a decision on publication. We will need to consider your response to these concerns in the form of a revised manuscript accompanied by a list to explain your revisions. You will also need to make some editorial changes to your paper so that it is as brief as possible and complies with our Guide to Authors (https://www.nature.com/nature/for-authors).

We extend our thanks to the recently retired Dr. Sage for handling the initial submission of our manuscript, and to Dr. Chadayammuri for the consideration of our revised manuscript for publication in *Nature*.

We have fully addressed the points raised by the two referees and have completed the requested editorial changes as detailed below.

Referee 1 recommends adding two Extended Data figures, which would bring the total to 11. Our nominal limit is ten, but we can allow 11 (you have nine right now).

Our revised manuscript contains 8 Extended Data Figures and 3 Extended Data Tables after implementing the recommended additions from Referee 1.

Please note that the presentation of a Nature paper has undergone significant changes in recent years to improve its readability and navigability online. Specifically, in most instances any supplementary text and data figures/tables can now be integrated into the main paper rather than presented in a separate Supplementary Information file. An overview of the key features and differences may be found in the Composition of a Nature Paper (http://s3-service-broker-live-19ea8b98-4d41-4cb4-be4c-d68f4963b7dd.s3.amazonaws.com/uploads/ckeditor/attachments/7824/3g_Paper_composition.pdf).

We now follow the standard manuscript structure of a Nature Paper.

This journal strongly supports public availability of data and custom code. Please place the data and code used in your paper into a public data repository, or alternatively, present the data as Supplementary Information. If data and code can only be shared on request, please explain why in your Data Availability Statement, and also in the correspondence with your editor. Please note that for some data types, deposition in a public repository is mandatory - more information on our data deposition policies and available repositories appears below.

All reduced transmission spectra data products will be publicly available in a Zenodo repository upon publication of the manuscript (the repository will be timed for public release in accordance with the standard embargo). We will also provide the specific atmospheric retrieval setup code used to run the open source POSEIDON package on our WD 1856b spectra in this Zenodo repository.

LENGTH: We estimate the current length of your paper to be ~1850 words, which exceeds our usual limit by a considerable margin. With four display items as at present, the main text of the revised version should be no more than ~1500 words. Keep in mind that important technical details that are not central to the main message of the paper can be moved into the Methods section or, if necessary, a Supplementary Information section (see below).

We have shortened the Main text to be less than 1500 words.

TITLES: Titles cannot exceed 75 characters (including spaces); they must not contain punctuation. We prefer that they convey the actual scientific bottom line of the paper, rather than simply indicate the topic (which is all that the present one does).

We have changed the title of our manuscript to reflect the main scientific results.

SUMMARY PARAGRAPH: All Nature papers begin with a fully referenced paragraph, typically no longer than 200 words. This paragraph starts with a 2- to 3-sentence summary of the state of the art immediately prior to this work. This should be *specific* -- statements like "the mechanism for orbital evolution ... is debated" is too vague. What are the options? Is there a

clear favorite? If so, why? As one of your main conclusions relates to orbital evolution, the background needs to complete. The paragraph continues with a 1-sentence statement of the main findings starting with 'Here we report' or an equivalent phrase. It concludes with 2 to 3 sentences putting the main findings into the context established earlier so it is clear how the results described in the paper have moved the field forward. In some cases it may be necessary to exceed this limit in order to explain complex material for readers in other fields – in such cases, summary paragraphs can be up to 230 words in length. The extra length, however, is for introduction and context, and not for additional technical information.

We have shortened the abstract to less than 230 words and adopted the specified format.

MAIN TEXT: **Further introductory material in the main text of the paper should not be necessary.** Any critical points made at lines 66-78 should be part of the background in the summary paragraph, with the rest omitted.

We have merged the introductory material with the abstract, with the Main text proceeding to present details of the observations of WD 1856b (following a similar format to other recently published *Nature* papers).

Any discussion at the end of the paper should also be brief, and not repeat what is already written in the initial summary paragraph.

We have cut down the discussion.

STATISTICS: Authors should ensure that any statistical analysis used is sound and that it conforms to the journal's guidelines (see https://www.nature.com/nature/for-authors/formatting-guide for guidance).

In addressing the referee points above, we have provided additional specificity on the statistics. In particular, residuals panels (in units of the standard deviation) are now included in the Figures.

METHODS: At the end of the main text document (after the main figure legends), there should be a section entitled "Methods", which provides a more detailed discussion of the additional methodological information that would allow other researchers to replicate the results (we define "Methods" quite broadly, so this is not limited to details of experimental protocols – supplementary discussion and analysis can also be included). The Methods section will not appear in the print version but will be fully copy-edited and appear online in the full-text HTML and PDF versions. The Methods section should be written as concisely as possible but should contain all elements necessary to allow interpretation and reproduction of the results. If there are additional references in the Methods section, their numbering should continue from the last reference in the main paper, and the list should follow the Methods section. If the methods require chemical structures, figures or tables, these should be supplied as Extended Data (see below). For mathematically complex methods, or methods that require an unusually large

number of figures or tables (beyond what can be accommodated as Extended Data), the entire Methods section should instead be supplied as a separate Supplementary Information.

We have split the Methods references from the Main text references as requested.

REFERENCES: As a guideline, most papers should need no more than 30 references in the main text; additional references can be cited in (and listed after) the Methods section, as detailed above. Please omit the arxiv numbers for any published papers.

The Main text has 28 references, in line with this requirement.

MAIN TEXT STATEMENTS: We require authors to provide a detailed Author Contribution statement immediately after the acknowledgements; the specific contributions of each author must be listed. It is also a condition of publication that authors include an Author Information statement indicating how to access information regarding reprints and permissions, stating whether or not there is a financial or non-financial competing interest, and naming the author to whom correspondence and requests for materials should be addressed. Please ensure that this section is included in the manuscript file after the Methods (but before the Extended Data legends) - it will not appear in the print version but will appear online in the full-text HTML and PDF versions. For details of "end note" style and an example see https://www.nature.com/nature/for-authors/formatting-guide.

We have verified that the Author Contribution statement and Author Information statements are complete.

DATA AVAILABILITY STATEMENT: All published manuscripts reporting original research in Nature Portfolio journals must include a data availability statement. The data availability statement must make the conditions of access to the “minimum dataset” that are necessary to interpret, verify and extend the research in the article, transparent to readers. This minimum dataset may be provided through deposition in public community/discipline-specific repositories, custom proprietary repositories for certain types of datasets, or general repositories like Figshare, Zenodo and Dryad. Providing large datasets in supplementary information is strongly discouraged and the preferred approach is to make data available in repositories. More information on Nature Portfolio’s reporting standards and preparing your Data Availability Statement can be found here: https://www.nature.com/nature-portfolio/editorial-policies/reporting-standards#reporting-requirements

The Data Availability statement is complete. As mentioned above, our final transmission spectra will be available in a Zenodo repository upon publication.

For all studies using custom code or mathematical algorithm that is deemed central to the conclusions, a statement must be included under the heading "Code availability", indicating whether and how the code or algorithm can be accessed, including any restrictions to access.

Code availability statements should be provided as a separate section after the data availability statement but before the References. Code should be deposited in a DOI-minting repository such as Zenodo, Gigantum or Code Ocean and cited in the reference list. Authors are encouraged to manage subsequent code versions and to use a license approved by the open source initiative. Additional details can be found here: https://www.nature.com/nature-research/editorial-policies/reporting-standards#availability-of-computer-code.

The Code Availability statement is complete.

FIGURE LEGENDS: The main figure legends should be listed sequentially after the references in the main text. Each legend should begin with a brief title for the whole figure and continue with a short description of each panel and the symbols used. Any error bars in the figures must be defined (for example, s.d., s.e.m.) and the value of n indicated; see https://www.nature.com/nature/for-authors/formatting-guide for further explanation. I know that 1sigma is the default in astronomy, but different fields use different defaults so we require authors to specify what they use.

We provide two versions of our manuscript: (i) the standard Nature format for Figure legends requested above and (ii) inline Figures for the referees.

DISPLAY ITEMS: Figures should be comprehensible to readers in other or related disciplines, and assist their understanding of the paper. We encourage authors who are describing complex processes to include a schematic of the main finding as part of the Extended Data to aid readers unfamiliar with the immediate discipline. Figures should be as small and simple as is compatible with clarity. All panels of a figure should be logically connected; each panel of a multipart figure should be sized so that the whole figure can be reduced by the same amount and reproduced on the printed page at the smallest size at which essential details are visible. For guidance, Nature's standard figure sizes are 89 mm (one column), 120 mm (one and a half columns), or exceptionally 183 mm (two columns) wide; the full depth of a Nature page is 247 mm. All panels of figures should be presented on a single page and assembled into a rectangular shape for publication; please indicate any essential alignments (parts horizontal, vertical, spacings of stereo pairs, etc.).

All Figures are provided on single pages at the end of the manuscript, as requested.

FIGURE FORMATTING: Lettering in all figures (labelling of axes and so on) should be in uniform, sans-serif font, in lower-case type, and large enough to permit substantial reduction for publication (minimum font size 5 pt). Separate parts of a figure are labelled a, b, etc. Units have a single space between the number and the unit, and follow SI nomenclature or the nomenclature common to a particular field. Thousands are separated by commas (1,000). Unusual units or abbreviations are defined in the legend. Scale bars rather than magnification factors should be used.

Our Figures adopt these formatting requirements for multiple panels etc.

IMAGE PRESENTATION: Authors should be aware that any image provided for publication, either in print or online (as Extended Data or Supplemental Information), may be subject to a quality control process to check for image integrity and manipulation. For a full discussion of our standards regarding how images should be prepared and presented, see www.nature.com/authors/editorial_policies/image.html.

No images are present in our manuscript.

EXTENDED DATA: Nature is now integrating the supplemental figures and tables into the final version of most papers. Extended Data do not appear in the printed version of the paper but are included online within the full-text HTML and at the end of the online PDF. Extended Data are an integral part of the paper and only data that directly contribute to the main message should be presented. All Extended Data must be referred to in the main text, figure legends and/or Methods section, and their figure legends should be listed sequentially at the end of the main text, not in the Extended Data files. Authors should assemble the Extended Data into a maximum of ten, A4 size, multi-panelled display items, submitted as individual JPEG, TIFF or EPS files. They must be provided at the same quality as figures for print, but there are important differences in their formatting. More specific instructions are provided in the Extended Data Formatting Guide (http://s3-service-broker-live-19ea8b98-4d41-4cb4-be4c-d68f4963b7dd.s3.amazonaws.com/uploads/ckeditor/attachments/7823/3h_Extended_data.pdf).

All Extended Data Figures and Tables are provided at the end of the manuscript.

SUPPLEMENTARY INFORMATION: Supplementary Information is online-only material published with the manuscript (https://www.nature.com/nature/for-authors/supp-info). For most papers, there should be no need for Supplementary Information beyond that already provided as Methods and Extended Data, the aim being to avoid unnecessary fragmentation of the paper online. Exceptions to this rule include large datasets that cannot be accommodated within Extended Data; video material; and more complex "Supplemental Methods" (and any associated references) that do not readily fit within the constraints of the Methods/Extended Data formats.
Please note that after the paper has been formally accepted you can only provide amended Supplementary Information files for critical changes to the scientific content, not for style. You should clearly explain what changes have been made if you do resupply any such files.

There is no Supplementary Information associated with this manuscript.

SOURCE DATA: To further increase transparency, we encourage authors to provide, in spreadsheet form, the data underlying the graphical representations used in the figures. This is in addition to our well-established data-deposition policy for specific types of experiments and large datasets. Readers of the online manuscript will be able to access the Source Data directly

from the figure legend. Spreadsheets can only be submitted in .xls, .xlsx or .csv formats. One file per figure is permitted; thus, if there is a multi-panelled figure the Source Data for each panel should be clearly labeled in the csv/Excel file; alternatively the data for a figure can be included in multiple, clearly labeled sheets within an Excel file. File sizes of up to 30 MB are permitted; however, it is expected that the vast majority of Source Data files will be considerably smaller than this. When submitting these files with your manuscript, please select the file type “Source Data” and use the title field in the file description tab to indicate the figure to which the Source Data pertain.

We now provide Source Data for Figure 1 (transit light curve), Figures 2 and Extended Data Figures 2 (planetary transmission spectra), and Extended Data Figure 3 (white dwarf spectrum).

Nature is committed to improving transparency in authorship. As part of our efforts in this direction, we are now requesting that all authors identified as ‘corresponding author’ create and link their Open Researcher and Contributor Identifier (ORCID) with their account on the Manuscript Tracking System prior to acceptance. ORCID helps the scientific community achieve unambiguous attribution of all scholarly contributions. You can create and link your ORCID from the home page of the Manuscript Tracking System by clicking on ‘Modify my Springer Nature account’ and following the instructions in the link below. If you experience problems in linking your ORCID, please contact the Platform Support Helpdesk.

ORCIDs have been provided.

When revising your paper:

* Include a point-by-point response to our referees and to any editorial suggestions. We are committed to providing a fair and constructive peer-review process. Please do not hesitate to contact us if there are specific requests from the reviewers that you believe are technically impossible or unlikely to yield a meaningful outcome.

* Ensure it complies with our format requirements as set out at https://www.nature.com/nature/for-authors.

All correspondence should contain your Nature reference number in the subject line.

We hope to receive your revised paper within four weeks. If you cannot send it within this time, we will still be happy to reconsider your paper at a later date so long as nothing similar has been accepted for publication at Nature or published elsewhere in the meantime.

We look forward to hearing from you soon.

Yours sincerely

Dr Leslie Sage

Response to the Referees

We thank the referees for their additional comments on our manuscript. We have now addressed their final comments, as described below.

Referee #1:

I appreciate the authors' efforts to address my comments. Overall, the latest version of the manuscript is very good. However, I have two concerns arising from the revisions that I would like to see addressed before recommending the paper for publication:

1. There has recently been substantial debate within the community regarding the use of "sigmas" to report the statistical significance of a detection of a chemical species or other atmospheric component. In particular, the sigma significance obtained following Benneke & Seager 2013, while frequently used in the exoplanet atmospheres community, has been shown to provide an overly optimistic assessment of the detection significance. Recent papers have argued for recalibrating the N sigma value to e.g. (N-1) sigma (Schmidt et al 2025), or to abandon it entirely and use Bayes factors due to its potential to mislead (Kipping and Benneke 2025). The authors clearly state which method they use to calculate "detection significances" (they follow the relation described in Benneke and Seager 2013), and also show the Bayes factors between different models in Extended Data Table 3, which is very helpful. It is fine to quote N sigma values following this method, as long as it is described clearly. However, the sigma values for hydrocarbons, CH4, aerosols and thermal emission are provided in the first paragraph of the main text without appropriate context. Given the potential for these values to mislead without clear acknowledgment of their caveats, I suggest that they are omitted from the summary paragraph. If the authors wish to describe their confidence in the identification of certain components, they could perhaps convert Bayes factors to weak/moderate/strong evidence using the Jeffreys' scale. However, it is important to be aware that any such conversion to sigmas or another metric is somewhat arbitrary, and the nuances are often lost on most readers, so I urge caution in any such conversion.

We thank the referee for highlighting the ongoing community debate around the Bayes factor to sigma mapping (which began after this manuscript was submitted). We have now updated the first paragraph to quote the Bayes factors (expressed as the more accessible term "odds ratios") instead of the sigma values from the Benneke & Seager 2013 relation. We have also updated the main text to include the ln Bayes factors alongside the sigma values.

In the methods, we continue to present both the Bayes factor and the 'equivalent sigma' for continuity with the literature. However, we have added some additional context in the methods surrounding the ongoing debate about Bayes factor to sigma mapping and pointed the reader to Kipping & Benneke (2025):

“For consistency with the exoplanet literature, we also convert the Bayes factors between two nested models (e.g. our reference model and a model excluding CH_4) into an ‘equivalent detection significance', Nσ, using a standard relation (Benneke & Seager 2013). We note, however, that there are several caveats associated with Bayes factor to detection significance mapping, so our preferred statistic for model preference is the Bayes factor/odds ratio (see Kipping & Benneke 2025).

2. Page 2, L94, right column: I am pleased that the authors now include the reduced chi squared values for the best-fit model for each data reduction. The authors state that they achieve “an excellent fit to the data” for both reductions. However, the reduced chi squared is quite different for each reduction (1.03 for FIREFLy, 1.28 for Juniper). For 102 degrees of freedom, the standard deviation of the reduced chi squared distribution is approximately 0.14 (following Andrae et al 2010). This suggests that the best fit to the FIREFLy reduction is well within one standard deviation, whereas the best fit to the Juniper reduction is discrepant with the data by around two standard deviations. This does not significantly impact the results of the paper, since there is good agreement between the posterior distributions of the retrievals on both reductions, but the statement at L94 should be replaced with a brief discussion on the different quality of fit for each reduction.

We have edited this paragraph to highlight the slightly worse fit for the Juniper reduction by adding the following:

“The fit quality is slightly less good for Juniper (χ^2_ν = 1.28, indicating consistently with the data within 2σ for 102 degrees of freedom), but the retrieval results are nonetheless consistent between the two data reductions.”

Referee #2:

I read the revised version of the manuscript and the authors' response. The authors have responded to all of my comments, and I have no further suggestions.

Note that the caption for Extended Data Fig 2 still refers to "Eureka! (pink squares)" even though those reductions were taken out of the paper.

We thank the referee for catching this and have ensured that there are no lingering mentions of Eureka! in the manuscript.
